# Efficacy and *in vitro* activity of gepotidacin against bacterial uropathogens, including drug-resistant phenotypes, in females with uncomplicated urinary tract infections: results from two global, pivotal, phase 3 trials (EAGLE-2 and EAGLE-3)

**DOI:** 10.1128/aac.01640-24

**Published:** 2025-09-09

**Authors:** Nicole E. Scangarella-Oman, Deborah L. Butler, John Breton, Derrek Brown, Cara Kasapidis, Helen Millns, Chun Huang, Caroline R. Perry, Amanda J. Sheets, Jeremy Dennison, Salim Janmohamed

**Affiliations:** 1 GSK 33139 , Collegeville, Pennsylvania, USA; 2 GSK 682071 , Stevenage, Hertfordshire, United Kingdom; 3 GSK 1929 , London, United Kingdom; University of Pittsburgh School of Medicine, Pittsburgh, Pennsylvania, USA

**Keywords:** gepotidacin, uncomplicated urinary tract infection, acute cystitis, uropathogen, phenotype, antibacterial resistance, nitrofurantoin, extended-spectrum β-lactamase, multidrug resistant

## Abstract

Two recent Phase 3 trials demonstrated the efficacy of gepotidacin compared with nitrofurantoin to treat uncomplicated urinary tract infections (uUTIs) in females. Pretreatment urine specimens were obtained from all participants. Based on pooled trial data (treatment groups combined), central laboratory culture results identified 1,421 (45%) participants with ≥1 baseline qualifying (≥10^5^ CFU/mL) uropathogen (i.e., pooled microbiological Intent-to-Treat population). *Escherichia coli*, *Klebsiella pneumoniae*, and *Proteus mirabilis* were the most common qualifying uropathogens. Among 1,159 *E. coli* isolates, 28%, 28%, 15%, and 28% were fluoroquinolone resistant (FQ-R), trimethoprim-sulfamethoxazole resistant (SXT-R), extended-spectrum β-lactamase positive (ESBL+), and multidrug resistant (MDR), respectively. For 114 *K*. *pneumoniae* isolates, 25%, 23%, 16%, and 16% were nitrofurantoin resistant, SXT-R, FQ-R, and ESBL+, respectively. Of 67 *P*. *mirabilis* isolates, 25%, 21%, and 31% were SXT-R, FQ-R, and MDR, respectively. Gepotidacin MIC_90_ values against all qualifying uropathogens and drug-resistant phenotypes ranged from 0.25 to 32 µg/mL, with no isolates of Enterobacterales, *Staphylococcus saprophyticus*, or *Enterococcus faecalis* considered resistant to gepotidacin. For all uropathogen drug-resistant phenotypes, gepotidacin MIC_90_ values were similar (i.e., lower, equal to, or 1-dilution higher) compared with the MIC_90_ of the overall species. Gepotidacin’s efficacy, based on therapeutic, clinical, and microbiological success rates, was generally consistent across phenotypic subgroups of *E. coli*, *K. pneumoniae*, and *P. mirabilis*. This pooled analysis represents a robust, contemporary, clinically relevant, and unbiased (i.e., baseline urine specimens obtained from all enrolled participants regardless of uUTI history) collection of data from community-acquired uUTIs in females.

**CLINICAL TRIALS:**

This study is registered with ClinicalTrials.gov as NCT04020341 and NCT04187144.

## INTRODUCTION

Urinary tract infections (UTIs) are among the most common bacterial infections worldwide (1). An estimated 50%–60% of adult females will experience at least one UTI in their lifetime (2).

Causative uncomplicated UTI (uUTI; acute cystitis) uropathogens are typically Gram-negative and Gram-positive bacteria such as *Escherichia coli*, *Enterococcus* spp., *Proteus mirabilis*, *Klebsiella pneumoniae*, *Staphylococcus saprophyticus*, and Group B *Streptococcus* (1, 3). Of these, *E. coli* is the most prevalent uropathogen isolated in community-acquired uUTIs and accounts for approximately 75% of infections overall (4–7). These uropathogens have diverse virulence factors and evolving mechanisms that lead to increased antibacterial resistance to current uUTI therapies (1). Over time, the viability of current antibiotic treatment options is expected to decline (8, 9), as uropathogens expand their array of resistance mechanisms. To battle evolving antimicrobial resistance among uropathogens, new oral antibiotics with novel mechanisms of action are needed to support outpatient uUTI treatment.

Gepotidacin was evaluated for the treatment of uUTIs in two recently completed global Phase 3 trials, EAGLE-2 (204989) and EAGLE-3 (212390) (10). Gepotidacin is a novel, bactericidal, first-in-class triazaacenaphthylene antibacterial that inhibits bacterial DNA replication by a unique mechanism of action, distinct binding site (11, 12), and provides well-balanced inhibition of two different type II topoisomerases (for most uUTI uropathogens) (13, 14). This provides activity against most strains of target uropathogens, such as *E. coli* and *S. saprophyticus*, including isolates resistant to current antibacterials (15, 16). Due to the well-balanced binding at both enzymes (i.e., gyrase and topoisomerase IV), a gepotidacin target-specific mutation in a single enzyme does not significantly affect gepotidacin susceptibility (13).

Based on the results of EAGLE-2 and EAGLE-3 (10), gepotidacin was approved in March 2025 by the US Food and Drug Administration (FDA) for the treatment of female adult and pediatric patients 12 years of age and older weighing at least 40 kg with uUTIs caused by the following susceptible microorganisms: *E. coli*, *K. pneumoniae*, *Citrobacter freundii* complex, *S. saprophyticus*, and *E. faecalis* (17).

As previously reported, in each Phase 3 trial, gepotidacin (1,500 mg twice daily for 5 days) was noninferior to nitrofurantoin (100 mg twice daily for 5 days), a standard-of-care first-line treatment agent, for the treatment of uUTI in a microbiologically confirmed nitrofurantoin-susceptible analysis population (10). Furthermore, the statistical superiority of gepotidacin to nitrofurantoin was also observed in EAGLE-3. Efficacy was determined based on combined clinical and microbiological response (i.e., therapeutic response) at the test-of-cure (TOC; days 10–13) visit. The primary analysis population only included participants with one or two baseline qualifying (growth ≥10^5^ CFU/mL) uropathogens that were susceptible to nitrofurantoin per Clinical and Laboratory Standards Institute (CLSI) guidelines (10, 18, 19). The safety and tolerability profiles of gepotidacin were acceptable in both trials (10).

Herein, pooled microbiology results and efficacy observations from the global EAGLE-2 and EAGLE-3 trials are presented with a focus on the overall incidence of recovered drug-resistant uropathogens, gepotidacin *in vitro* activity against drug-resistant phenotypes, and by-pathogen efficacy responses against drug-resistant phenotypes. Data are based primarily on the microbiological Intent-to-Treat (micro-ITT) population, which included all randomized participants who received at least one dose of study treatment and had a baseline qualifying (growth ≥10^5^ CFU/mL) uropathogen regardless of its susceptibility to nitrofurantoin (10). This population includes a higher number of participants and uropathogen data for assessments in contrast to the primary analysis population, which only included micro-ITT participants with nitrofurantoin-susceptible baseline qualifying uropathogens (10), thus providing a broader and more clinically relevant data set.

These pooled EAGLE-2 and EAGLE-3 uropathogen data are distinct from other data sets for community-acquired uUTIs as they are based on urine samples collected from all participants without selection bias. Other databases contain cultures typically performed for selected patients based on past medical history or recurrence of uUTIs, whereas our approach has led to a comprehensive and unbiased set of contemporary uropathogen prevalence data in females with uUTIs. In a co-publication, the incidence of drug-resistant and epidemiological genotypes/clones in a subset of the pooled uropathogen data and gepotidacin’s *in vitro* activity and efficacy for the genotypes are presented (20).

## RESULTS

### Uropathogens and phenotypic subcategories

In the pooled ITT population, 1,421 (45%) participants had at least one baseline qualifying (≥10^5^ CFU/mL) uropathogen recovered and were included in the pooled micro-ITT population for the combined treatment groups (Table 1). Among these micro-ITT participants, 1,486 baseline qualifying uropathogens were recovered; the most common identified was *E. coli* (78%), followed by *K. pneumoniae* (8%), *P. mirabilis* (5%), and *S. saprophyticus* (2%). All other baseline qualifying uropathogens identified in the pooled micro-ITT population had an incidence of ≤1%. A summary of all baseline uropathogens (≥10^3^ CFU/mL) identified in the ITT population is provided in Table S1.

**TABLE 1:** Incidence of baseline qualifying uropathogens recovered and selected drug-resistant phenotypes for pooled EAGLE-2 and EAGLE-3 data (micro-ITT population)

Qualifying uropathogen/phenotype or combined phenotypes^*b*^	Treatment group		Total n (%)*^a^* *N* = 1,421
	Gepotidacin n (%)*^a^* *N* = 732	Nitrofurantoin n (%)*^a^* *N* = 689	
Total number of qualifying uropathogens recovered	764	722	1,486
*Escherichia coli*	598 (78)	561 (78)	1,159 (78)
Amoxicillin-clavulanic acid resistant*^c^*	29 (5)	20 (4)	49 (4)
Ampicillin resistant	289 (48)	261 (47)	550 (47)
Cefadroxil resistant^*d*^	95 (16)	73 (13)	168 (14)
Cefazolin resistant^*e*^	107 (18)	85 (15)	192 (17)
Ceftazidime-avibactam resistant	0	1 (<1)	1 (<1)
Ceftolozane-tazobactam resistant	4 (1)	6 (1)	10 (1)
Ceftriaxone resistant	85 (14)	71 (13)	156 (13)
Fosfomycin resistant^*f*^	5 (1)	4 (1)	9 (1)
FQ-R^*g*^	179 (30)	151 (27)	330 (28)
Gentamicin resistant	56 (9)	58 (10)	114 (10)
Mecillinam resistant	18 (3)	14 (2)	32 (3)
Nitrofurantoin resistant^*h*^	6 (1)	8 (1)	14 (1)
Nitroxoline resistant	12 (2)	12 (2)	24 (2)
Piperacillin-tazobactam resistant^*i*^	12 (2)	11 (2)	23 (2)
SXT-R	172 (29)	149 (27)	321 (28)
ESBL+^*j*^	94 (16)	76 (14)	170 (15)
MDR^*k*^	175 (29)	148 (26)	323 (28)
Fosfomycin resistant^*f*^ and ESBL *+^j^*	3 (1)	2 (<1)	5 (<1)
FQ-R^*g*^ and ESBL *+^j^*	70 (12)	53 (9)	123 (11)
FQ-R^*g*^ and ESBL*+^i^* and SXT-R	38 (6)	28 (5)	66 (6)
FQ-R^*g*^ and SXT-R	80 (13)	71 (13)	151 (13)
SXT-R and ESBL+^*j*^	54 (9)	36 (6)	90 (8)
*Klebsiella pneumoniae*	56 (7)	58 (8)	114 (8)
Amoxicillin-clavulanic acid resistant^*c*^	5 (9)	5 (9)	10 (9)
Cefadroxil resistant^*d*^	10 (18)	7 (12)	17 (15)
Cefazolin resistant^*e*^	10 (18)	8 (14)	18 (16)
Ceftolozane-tazobactam resistant	2 (4)	1 (2)	3 (3)
Ceftriaxone resistant	8 (14)	7 (12)	15 (13)
FQ-R^*g*^	9 (16)	9 (16)	18 (16)
Gentamicin resistant	2 (4)	4 (7)	6 (5)
Nitrofurantoin resistant	13 (23)	15 (26)	28 (25)
Piperacillin-tazobactam resistant	3 (5)	4 (7)	7 (6)
SXT-R	13 (23)	13 (22)	26 (23)
ESBL+^*j*^	11 (20)	7 (12)	18 (16)
MDR^*k*^	14 (25)	13 (22)	27 (24)
FQ-R^*g*^ and ESBL+^*j*^	8 (14)	6 (10)	14 (12)
FQ-R^*g*^ and ESBL+^*j*^ and SXT-R	3 (5)	4 (7)	7 (6)
FQ-R^*g*^ and SXT-R	3 (5)	4 (7)	7 (6)
SXT-R and ESBL+^*j*^	5 (9)	5 (9)	10 (9)
*Klebsiella oxytoca/Raoultella ornithinolytica*	5 (<1)	4 (<1)	9 (<1)
Cefazolin resistant	4 (80)	3 (75)	7 (78)
MDR^*k*^	1 (20)	1 (25)	2 (22)
*Klebsiella aerogenes*	2 (<1)	5 (<1)	7 (<1)
*Klebsiella variicola*	3 (<1)	2 (<1)	5 (<1)
Nitrofurantoin resistant	0	1 (50)	1 (20)
*Proteus mirabilis*	34 (4)	33 (5)	67 (5)
Ampicillin resistant	4 (12)	3 (9)	7 (10)
Cefadroxil resistant^*d*^	9 (26)	9 (27)	18 (27)
Cefazolin resistant^*e*^	3 (9)	0	3 (4)
Ceftriaxone resistant	2 (6)	0	2 (3)
FQ-R^*g*^	11 (32)	3 (9)	14 (21)
Gentamicin resistant	2 (6)	2 (6)	4 (6)
SXT-R	12 (35)	5 (15)	17 (25)
ESBL+^*j*^	2 (6)	0	2 (3)
MDR^*k*^	16 (47)	5 (15)	21 (31)
FQ-R^*g*^ and ESBL+^*j*^	1 (3)	0	1 (1)
FQ-R^*g*^ and ESBL+^*j*^ and SXT-R	1 (3)	0	1 (1)
FQ-R^*g*^ and SXT-R	10 (29)	3 (9)	13 (19)
SXT-R and ESBL+^*j*^	1 (3)	0	1 (1)
*Enterobacter cloacae* complex	6 (<1)	11 (2)	17 (1)
Ceftolozane-tazobactam resistant	0	1 (9)	1 (6)
Ceftriaxone resistant	0	3 (27)	3 (18)
Nitrofurantoin resistant	0	1 (9)	1 (6)
Piperacillin-tazobactam resistant	0	3 (27)	3 (18)
MDR^*k*^	6 (100)	11 (100)	17 (100)
*Citrobacter freundii* complex	13 (2)	6 (<1)	19 (1)
Amoxicillin-clavulanic acid resistant^*c*^	7 (54)	2 (33)	9 (47)
Ampicillin resistant	8 (62)	0	8 (42)
Ceftolozane-tazobactam resistant	2 (15)	0	2 (11)
Ceftriaxone resistant	5 (38)	0	5 (26)
FQ-R^*g*^	1 (8)	0	1 (5)
Piperacillin-tazobactam resistant	4 (31)	0	4 (21)
SXT-R	3 (23)	1 (17)	4 (21)
MDR^*k*^	8 (62)	0	8 (42)
FQ-R^*g*^ and SXT-R	1 (8)	0	1 (5)
*Citrobacter koseri*	2 (<1)	6 (<1)	8 (<1)
*Citrobacter amalonaticus* group	2 (<1)	0	2 (<1)
Cefazolin resistant	1 (50)	0	1 (50)
FQ-R^*g*^	1 (50)	0	1 (50)
*Morganella morganii*	8 (1)	5 (<1)	13 (<1)
Ceftriaxone resistant	1 (13)	0	1 (8)
FQ-R^*g*^	1 (13)	2 (40)	3 (23)
Gentamicin resistant	1 (13)	0	1 (8)
SXT-R	2 (25)	0	2 (15)
MDR^*k*^	8 (100)	5 (100)	13 (100)
*Serratia marcescens*	0	3 (<1)	3 (<1)
Ceftazidime-avibactam resistant	0	1 (33)	1 (33)
Ceftolozane-tazobactam resistant	0	1 (33)	1 (33)
Ceftriaxone resistant	0	1 (33)	1 (33)
FQ-R^*g*^	0	1 (33)	1 (33)
Meropenem resistant	0	1 (33)	1 (33)
Piperacillin-tazobactam resistant	0	1 (33)	1 (33)
SXT-R	0	1 (33)	1 (33)
MDR^*k*^	0	3 (100)	3 (100)
*Providencia rettgeri*	1 (<1)	1 (<1)	2 (<1)
MDR^*k*^	1 (100)	1 (100)	2 (100)
*Serratia liquefaciens*	1 (<1)	0	1 (<1)
Cefazolin resistant	1 (100)	0	1 (100)
*Pseudomonas aeruginosa*	2 (<1)	3 (<1)	5 (<1)
Cefazolin resistant	2 (100)	3 (100)	5 (100)
FQ-R^*g*^	1 (50)	0	1 (20)
MDR^*k*^	2 (100)	3 (100)	5 (100)
*Pseudomonas putida* group	1 (<1)	1 (<1)	2 (<1)
Cefazolin resistant	1 (100)	1 (100)	2 (100)
Gentamicin resistant	1 (100)	1 (100)	2 (100)
Meropenem resistant	1 (100)	1 (100)	2 (100)
Piperacillin-tazobactam resistant	1 (100)	1 (100)	2 (100)
SXT-R	1 (100)	1 (100)	2 (100)
MDR^*k*^	1 (100)	1 (100)	2 (100)
*Acinetobacter baumannii nosocomialis* group	0	1 (<1)	1 (<1)
Cefazolin resistant	0	1 (100)	1 (100)
*Weeksella virosa*	0	1 (<1)	1 (<1)
Cefazolin resistant	0	1 (100)	1 (100)
FQ-R^*g*^	0	1 (100)	1 (100)
*Staphylococcus saprophyticus*	15 (2)	14 (2)	29 (2)
Ampicillin resistant	4 (27)	4 (29)	8 (28)
Methicillin resistant	3 (20)	1 (7)	4 (14)
Penicillin resistant	9 (60)	8 (57)	17 (59)
MDR^*k*^	0 (0)	1 (7)	1 (3)
*Enterococcus faecalis*	14 (2)	7 (<1)	21 (1)
Fosfomycin resistant^*f*^	1 (7)	0	1 (5)
FQ-R^*g*^	4 (29)	2 (29)	6 (29)
Penicillin resistant	4 (29)	1 (14)	5 (24)
MDR^*k*^	3 (21)	1 (14)	4 (19)
FQ-R^*g*^ and SXT-R	4 (29)	2 (29)	6 (29)
*Enterococcus faecium*	1 (<1)	0	1 (<1)

^*a*^ Percentage of each qualifying uropathogen was calculated using the total number of baseline qualifying uropathogens at baseline as the denominator. The percentage of each phenotypic subcategory was calculated using the number of each respective baseline qualifying uropathogen as the denominator in a post hoc analysis.

^*b*^ Baseline qualifying (≥10^5^ CFU/mL) uropathogens and selected drug-resistant phenotypes are presented. All drug-resistant phenotypes were determined per CSLI or EUCAST guidelines as described in the footnotes. Phenotypes were determined per CLSI M100 2022 guidelines (19) with the exception of cefadroxil and nitroxoline, which were determined by EUCAST 2022 guidelines (21). Phenotypes that are 100% resistant due to intrinsic resistance of the organism to the drug per CLSI M100 2022 guidelines are not presented. Isolates with intermediate susceptibility interpretations were not included in the corresponding drug-resistant phenotypic categories.

^*c*^ Breakpoints for therapy of uUTIs; tested according to CLSI guidelines in a 2:1 ratio.

^*d*^ Tested by disk diffusion method; breakpoints and interpretations per EUCAST guidelines for uUTI only.

^*e*^ Breakpoints for when used as a surrogate test for oral cephalosporins for therapy of uUTIs due to *E. coli*, *K. pneumoniae*, and *P. mirabilis*.

^*f*^ Tested by agar dilution method using media supplemented with 25 µg/mL of glucose-6-phosphate; breakpoints apply only to urinary tract isolates.

^*g*^ FQ-R indicates resistance to ciprofloxacin and/or levofloxacin using CLSI breakpoints.

^*h*^ Tested by disk diffusion method; interpretations per CLSI guidelines for testing and reporting of *E. coli* urinary tract isolates only.

^*i*^ Tested by disk diffusion method; breakpoints and interpretations per EUCAST guidelines for uUTIs only due to *E. coli*.

^*j*^ ESBL+ indicates extended spectrum β-lactamase production per CLSI M100 Table 3A.

^*k*^ MDR was defined as resistance to ≥3 relevant antibacterial classes. *E. cloacae* complex and *M. morganii* MDR isolates are associated with intrinsic resistance.

Among 1,159 *E. coli* isolates in the pooled micro-ITT population, 28% were fluoroquinolone resistant (FQ-R), 28% were trimethoprim-sulfamethoxazole resistant (SXT-R), 15% were extended spectrum β-lactamase positive (ESBL+), and 28% were MDR (Table 1). With the exception of SXT, the percentage of *E. coli* isolates with drug-resistant phenotypes to first-line recommended treatments was low; 1%, 1%, and 3% were fosfomycin resistant, nitrofurantoin resistant, and mecillinam resistant (i.e., pivmecillinam resistant), respectively. The percentage of cephalosporin-resistant *E. coli* phenotypes ranged from 13% to 17%, not including the cephalosporin-β-lactamase inhibitor combinations. No baseline qualifying *E. coli* isolates were meropenem resistant.

For the 114 baseline qualifying *K. pneumoniae* isolates in the pooled micro-ITT population, the most common drug-resistant phenotypes were nitrofurantoin resistant (25%) and SXT-R (23%), followed by FQ-R and ESBL+ isolates (16% each) (Table 1). The percentage of cephalosporin-resistant *K. pneumoniae* ranged from 13% to 16% when excluding the cephalosporin-*β-*lactamase inhibitor combinations. Of the *K. pneumoniae* isolates, 24% were MDR. None of the baseline qualifying *K. pneumoniae* isolates were meropenem resistant.

There were 67 baseline qualifying *P. mirabilis* isolates in the pooled micro-ITT population (Table 1). Of these, 25% were SXT-R, 21% were FQ-R, and 31% were MDR. A higher percentage of cefadroxil-resistant *P. mirabilis* isolates (27%) were observed compared with ceftriaxone- and cefazolin-resistant isolates (3% and 4%, respectively).

Overall, 15% of pooled micro-ITT participants had a baseline qualifying uropathogen that was not susceptible to nitrofurantoin, whereas 85% of participants had nitrofurantoin-susceptible (NTF-S) uropathogens and comprised the pooled micro-ITT NTF-S population (i.e., the population in which gepotidacin’s efficacy was demonstrated [10]). For reference, the incidence of baseline qualifying uropathogens recovered and selected drug-resistant phenotypes for the pooled micro-ITT NTF-S population is presented in Table S2.

### *In vitro* activity of gepotidacin against phenotypic subcategories

Based on recently established FDA breakpoints and interpretive criteria for gepotidacin (22), the vast majority of Enterobacterales isolates were susceptible (MICs ≤ 16 µg/mL) and none were resistant (MIC ≥64 µg/mL) to gepotidacin for baseline uropathogen species and drug-resistant phenotypes recovered in the micro-ITT population (Table 2).

**TABLE 2:** Gepotidacin MIC frequency distribution against selected baseline qualifying uropathogens and drug-resistant phenotypes with *n* ≥10 isolates across both treatment groups for pooled EAGLE-2 and EAGLE-3 data (micro-ITT population)

Uropathogen/phenotypic subcategory^*a*^	No. of isolates^*b*^	No. of isolates/(cumulative %) inhibited with gepotidacin MIC (µg/mL) of^*c*^											MIC_50_	MIC_90_
		≤0.03	0.06	0.12	0.25	0.5	1	2	4	8	16	32	µg/mL	µg/mL
*Escherichia coli*	1,159	1 (<0.1)	0 (<0.1)	2 (0.3)	11 (1.2)	101 (9.9)	502 (53.2)	424 (89.8)	93 (97.8)	17 (99.3)	7 (>99.9)	1 (100)	1	4
Amoxicillin-clavulanic acid resistant^*d*^	49					3 (6.1)	17 (40.8)	16 (73.5)	10 (93.9)	2 (98.0)	1 (100)		2	4
Ampicillin resistant	550			2 (0.4)	9 (2.0)	49 (10.9)	233 (53.3)	186 (87.1)	52 (96.5)	12 (98.7)	6 (99.8)	1 (100)	1	4
Cefadroxil resistant^*e*^	168			1 (0.6)	2 (1.8)	18 (12.5)	55 (45.2)	60 (81.0)	20 (92.9)	9 (98.2)	3 (100)		2	4
Cefazolin resistant^*f*^	192			2 (1.0)	2 (2.1)	18 (11.5)	68 (46.9)	68 (82.3)	22 (93.8)	8 (97.9)	4 (100)		2	4
Ceftriaxone resistant	156			1 (0.6)	2 (1.9)	15 (11.5)	52 (44.9)	56 (80.8)	18 (92.3)	8 (97.4)	4 (100)		2	4
FQ-R^*g*^	330	1 (0.3)	0 (0.3)	2 (0.9)	8 (3.3)	59 (21.2)	116 (56.4)	96 (85.5)	39 (97.3)	6 (99.1)	3 (100)		1	4
Mecillinam resistant^*h*^	32				1 (3.1)	2 (9.4)	10 (40.6)	11 (75.0)	6 (93.8)	1 (96.9)	0 (96.9)	1 (100)	2	4
Nitrofurantoin resistant	14					2 (14.3)	4 (42.9)	4 (71.4)	4 (100)				2	4
Nitroxoline resistant^*i*^	24					1 (4.2)	10 (45.8)	7 (75.0)	3 (87.5)	3 (100)			2	8
SXT-R	321			1 (0.3)	4 (1.6)	37 (13.1)	152 (60.4)	95 (90.0)	23 (97.2)	6 (99.1)	3 (100)		1	2
ESBL+^*j*^	170			1 (0.6)	2 (1.8)	17 (11.8)	59 (46.5)	60 (81.8)	19 (92.9)	8 (97.6)	4 (100)		2	4
MDR^*k*^	323			2 (0.6)	4 (1.9)	38 (13.6)	125 (52.3)	103 (84.2)	38 (96.0)	10 (99.1)	3 (100)		1	4
FQ-R^*g*^ and ESBL+^*d*^	123			1 (0.8)	1 (1.6)	14 (13.0)	39 (44.7)	47 (82.9)	15 (95.1)	3 (97.6)	3 (100)		2	4
FQ-R^*g*^ and ESBL+^*d*^ and SXT-R	66			1 (1.5)	1 (3.0)	10 (18.2)	23 (53.0)	24 (89.4)	5 (97.0)	0 (97.0)	2 (100)		1	4
FQ-R^*g*^ and SXT-R	151			1 (0.7)	4 (3.3)	24 (19.2)	60 (58.9)	43 (87.4)	15 (97.4)	2 (98.7)	2 (100)		1	4
SXT-R and ESBL+^*j*^	90			1 (1.1)	1 (2.2)	12 (15.6)	33 (52.2)	31 (86.7)	8 (95.6)	2 (97.8)	2 (100)		1	4
*Klebsiella pneumoniae*	114						1 (0.9)	13 (12.3)	53 (58.8)	34 (88.6)	9 (96.5)	4 (100)	4	16
Amoxicillin-clavulanic acid resistant^*b*^	10								5 (50.0)	2 (70.0)	2 (90.0)	1 (100)	4	16
Cefadroxil resistant^*e*^	17							2 (11.8)	7 (52.9)	3 (70.6)	4 (94.1)	1 (100)	4	16
Cefazolin resistant^*f*^	18							2 (11.1)	8 (55.6)	3 (72.2)	4 (94.4)	1 (100)	4	16
Ceftriaxone resistant	15							2 (13.3)	6 (53.3)	3 (73.3)	4 (100)		4	16
FQ-R^*g*^	18							3 (16.7)	7 (55.6)	4 (77.8)	2 (88.9)	2 (100)	4	32
Nitrofurantoin resistant	28							3 (10.7)	13 (57.1)	8 (85.7)	3 (96.4)	1 (100)	4	16
SXT-R	26						1 (3.8)	1 (7.7)	10 (46.2)	9 (80.8)	3 (92.3)	2 (100)	8	16
ESBL+^*d*^	18							2 (11.1)	7 (50.0)	3 (66.7)	4 (88.9)	2 (100)	4	32
MDR^*k*^	27							2 (7.4)	10 (44.4)	9 (77.8)	4 (92.6)	2 (100)	8	16
FQ-R^*g*^ and ESBL+^*j*^	14							2 (14.3)	6 (57.1)	2 (71.4)	2 (85.7)	2 (100)	4	32
SXT-R and ESBL+^*j*^	10							1 (10.0)	4 (50.0)	2 (70.0)	3 (100)		4	16
*Proteus mirabilis*	67						3 (4.5)	4 (10.4)	15 (32.8)	31 (79.1)	11 (95.5)	3 (100)	8	16
Cefadroxil resistant^*e*^	18							1 (5.6)	4 (27.8)	9 (77.8)	2 (88.9)	2 (100)	8	32
FQ-R^*g*^	14						3 (21.4)	3 (42.9)	5 (78.6)	3 (100)			4	8
SXT-R	17						2 (11.8)	3 (29.4)	5 (58.8)	6 (94.1)	1 (100)		4	8
MDR^*k*^	21						3 (14.3)	3 (28.6)	6 (57.1)	8 (95.2)	1 (100)		4	8
FQ-R^*g*^ and SXT-R	13						2 (15.4)	3 (38.5)	5 (76.9)	3 (100)			4	8
*Enterobacter cloacae complex*	17							3 (17.6)	14 (100)				4	4
*Citrobacter freundii complex*	19					2 (10.5)	6 (42.1)	5 (68.4)	4 (89.5)	2 (100)			2	8
*Morganella morganii*	13						2 (15.4)	2 (30.8)	5 (69.2)	2 (84.6)	2 (100)		4	16
*Staphylococcus saprophyticus*	29		11 (37.9)	14 (86.2)	2 (93.1)	2 (100)							0.12	0.25
*Enterococcus faecalis*	21					2 (9.5)	11 (61.9)	6 (90.5)	2 (100)				1	2

^*a*^ All drug-resistant phenotypes were determined per CSLI or EUCAST guidelines as described in the footnotes. Phenotypes were determined per CLSI M100 2022 guidelines (19) with the exception of cefadroxil and nitroxoline, which were determined by EUCAST 2022 guidelines (21). Phenotypes are not presented if they are 100% resistant due to intrinsic resistance of the organism to the drug per CLSI M100 2022 guidelines. *S. saprophyticus* and *E. faecalis* drug-resistant phenotypes are not presented as the only phenotypes with *n* ≥10 isolates that are 100% resistant due to intrinsic resistance (fosfomycin resistant and SXT-R, respectively). Isolates with intermediate susceptibility interpretations were not included in the corresponding drug-resistant phenotypic categories.

^*b*^ Number of isolates with non-missing MIC values.

^*c*^ The percentage of isolates shown at each gepotidacin MIC concentration was calculated as a cumulative value in a post hoc analysis. Gepotidacin MIC values ranged from ≤0.03 to 32 µg/mL; MIC columns for 64 µg/mL and >64 µg/mL are not shown as there were no data to present.

^*d*^ Breakpoints for therapy of uUTIs; tested according to CLSI guidelines in a 2:1 ratio.

^*e*^ Tested by disk diffusion method. Breakpoints and interpretations per EUCAST guidelines for uUTIs only.

^*f*^ Breakpoints for when used as a surrogate test for oral cephalosporins for therapy of uUTIs due to *E. coli* and *K. pneumoniae*.

^*g*^ FQ-R indicates resistance to ciprofloxacin and/or levofloxacin using CLSI breakpoints.

^*h*^ Tested by disk diffusion method. Interpretations were per CLSI guidelines for testing and reporting of *E. coli* urinary tract isolates only.

^*i*^ Tested by disk diffusion method. Breakpoints and interpretations per EUCAST guidelines for uUTI only due to *E. coli*.

^*j*^ ESBL+ indicates extended spectrum β-lactamase production per CLSI M100 Table 3A.

^*k*^ MDR was defined as resistance to ≥3 relevant antibacterial classes.

In the micro-ITT population, the gepotidacin MIC_90_ value was 4 µg/mL against *E. coli* (Table 2). Overall, >99.9% of *E. coli* isolates, and 100% of FQ-R, SXT-R, nitrofurantoin-resistant, ESBL+, and MDR *E. coli* isolates were susceptible to gepotidacin (Table 2) (22). Only for nitroxoline-resistant *E. coli* isolates, a gepotidacin MIC_90_ of 8 µg/mL was observed, which was 1-dilution higher compared with *E. coli* isolates overall (4 µg/mL); 100% of isolates were susceptible to gepotidacin. The percentage of *E. coli* isolates susceptible to gepotidacin for other drug-resistant phenotypes ranged from 96.9% to 100%.

Against *K. pneumoniae* isolates, the gepotidacin MIC_90_ value was 16 µg/mL, including most drug-resistant phenotypes, and 96.5% of all isolates were susceptible to gepotidacin (Table 2) (22). Four *K. pneumoniae* isolates had intermediate susceptibility to gepotidacin with MIC values of 32 µg/mL. The gepotidacin MIC_90_ value was 1-dilution higher (32 µg/mL) against FQ-R, ESBL+, and the combined FQ-R and ESBL+ phenotype subcategories, with 88.9%, 88.9%, and 85.7% of isolates susceptible to gepotidacin, respectively. The percentage of *K. pneumoniae* isolates susceptible to gepotidacin for other drug-resistant phenotypes ranged from 85.7% to 100%.

Against *P. mirabilis* isolates, the gepotidacin MIC_90_ value was 16 µg/mL and 95.5% of isolates were susceptible to gepotidacin (Table 2) (22). Gepotidacin MIC_90_ values were lower (8 µg/mL) across most drug-resistant phenotypes, with the exception of cefadroxil-resistant *P. mirabilis* isolates that had a 1-dilution higher MIC_90_ (32 µg/mL; 88.9% of isolates were susceptible to gepotidacin). The percentage of *P. mirabilis* isolates susceptible to gepotidacin for other drug-resistant phenotypes was 100%. Among other Enterobacterales species, gepotidacin MIC_90_ values were 4 µg/mL (*Enterobacter cloacae* complex), 8 µg/mL (*Citrobacter freundii* complex), and 16 µg/mL (*Morganella morganii*), with 100% of isolates for each species susceptible to gepotidacin. Gepotidacin demonstrated activity against Gram-positive uropathogens, with MIC_90_ values of 0.25 µg/mL and 2 µg/mL for *S. saprophyticus* and *E. faecalis*, respectively; 93.1% of *S. saprophyticus* and 100% of *E. faecalis* isolates were susceptible to gepotidacin.

For reference, the *in vitro* activity of gepotidacin against the qualifying uropathogens and phenotypic subcategories in the pooled micro-ITT NTF-S population is presented in Table S3.

### Gepotidacin efficacy against phenotypic subcategories

**Fig 1:**
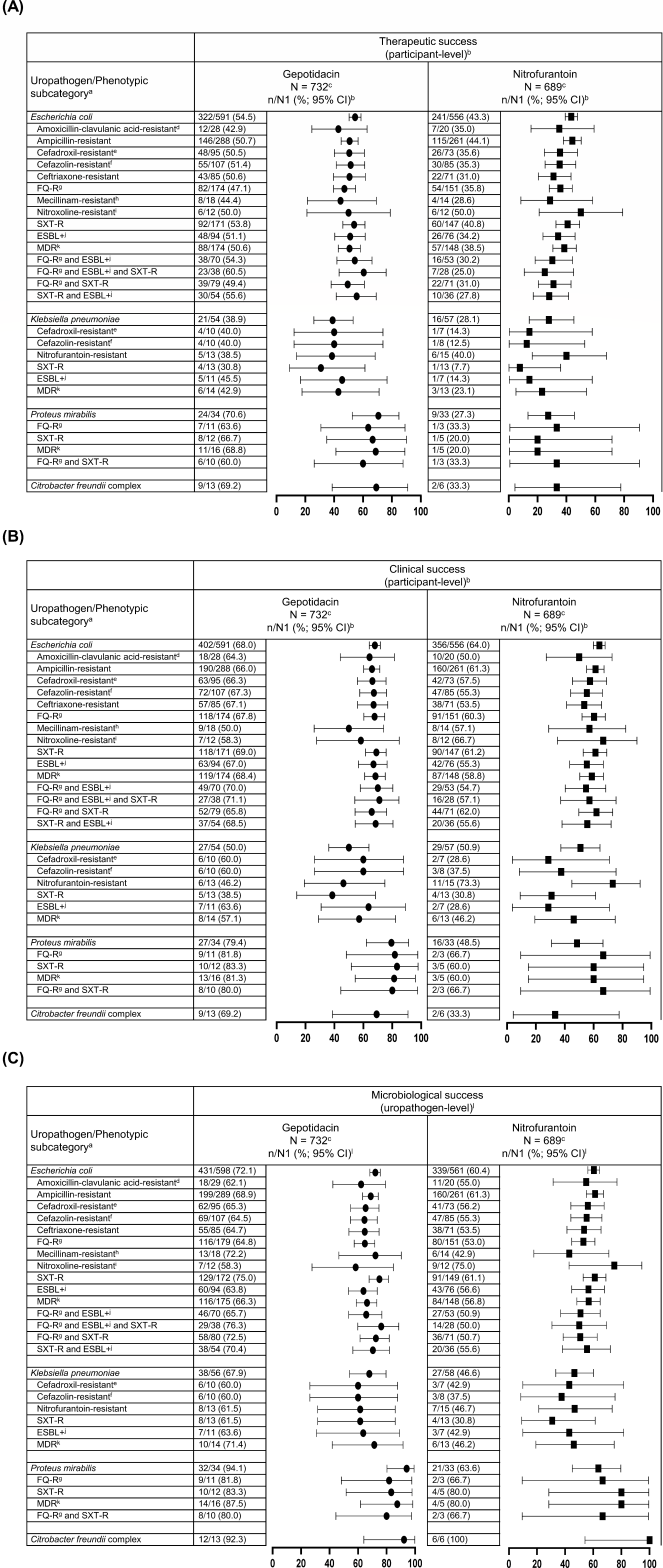
Therapeutic (**A**), clinical (**B**), and microbiological (**C**) success at TOC by selected baseline qualifying uropathogens and drug-resistant phenotypes with *n* ≥ 10 participants for at least one treatment group for pooled EAGLE-2 and EAGLE-3 data (micro-ITT population). *^a^*All drug-resistant phenotypes were per the CSLI or EUCAST guidelines as described in the footnotes. Phenotypes were determined per CLSI M100 2022 guidelines (19) with the exception of cefadroxil and nitroxoline, which were determined by EUCAST 2022 guidelines (21). Isolates with intermediate susceptibility interpretations were not included in the corresponding drug-resistant phenotypic categories. Only baseline qualifying uropathogen (present at ≥10^5^ CFU/mL) phenotypes with *n* ≥ 10 isolates for the pooled trials in at least one treatment group are displayed. Of note, additional phenotypes are presented in Table 2 due to the different threshold of ≥10 participants in the combined treatment groups. *^b^*For therapeutic and clinical response, a participant was counted once under a uropathogen category if multiple qualifying uropathogens within that category were isolated at baseline for the participant. Participants for whom all uropathogens were not eradicated and all symptoms were not resolved were considered therapeutic failures for all uropathogens. For therapeutic and clinical success: n/N1 = (n) the number of participants within the category with a response of success/(N1) the total number of participants within the category, and is the denominator for corresponding percentages. *^c^*The N in the header represents the total number of participants in the treatment arm for pooled data. *^d^*Breakpoints for therapy of uUTIs; tested according to CLSI guidelines in a 2:1 ratio. *^e^*Tested by the disk diffusion method. Breakpoints and interpretations per EUCAST guidelines for uUTIs only. *^f^*Breakpoints for when used as a surrogate test for oral cephalosporins for treatment of uUTIs due to *E. coli. ^g^*FQ-R resistance to ciprofloxacin and/or levofloxacin using CLSI breakpoints. *^h^*Tested by the disk diffusion method. Interpretations were per CLSI guidelines for testing and reporting of *E. coli* urinary tract isolates only. *^i^*Tested by the disk diffusion method. Breakpoints and interpretations per EUCAST guidelines for uUTIs only due to *E. coli. ^j^*ESBL+ indicates extended spectrum β-lactamase production per CLSI M100 Table 3A. *^k^*MDR was defined as resistance to ≥3 relevant antibacterial classes. *^l^*For microbiological response, a participant was counted more than once under a uropathogen category if multiple qualifying uropathogens within that category were isolated at baseline for the participant. For microbiological success: n/N1 = (n) the number of isolates that are a microbiological success/(N1) the total number of isolates in the category and is the denominator for the corresponding percentages.

Figure 1 presents therapeutic, clinical, and microbiological success at TOC by baseline qualifying uropathogen and selected drug-resistant phenotypes in the pooled micro-ITT population. Within the gepotidacin treatment group, therapeutic, clinical, and microbiological success rates were similar across phenotypic subgroups of *E. coli*. Though sample sizes were small for each *K. pneumoniae* drug-resistant phenotype (*n* ≤ 14) and *P. mirabilis* drug-resistant phenotypes (*n* ≤ 16), gepotidacin success rates for each endpoint were consistent with the corresponding success rates for each overall species. Generally, and as expected, success rates were numerically higher in the gepotidacin group compared with the nitrofurantoin group since this analysis population included isolates not susceptible to nitrofurantoin, particularly *K. pneumoniae* and *P. mirabilis*. Of note, similar gepotidacin efficacy trends were observed in the microbiologically evaluable (ME) population at TOC (ME-TOC population) and in the ME population at Follow-up (FU; ME-FU population) in Tables S4 and S5, respectively. This set of study participants met minimal dosing criteria, had non-missing outcome assessments at the respective visit, and had no other protocol deviation that confounded the efficacy assessments.

In the pooled micro-ITT NTF-S population, therapeutic, clinical, and microbiological success rates generally favored gepotidacin compared with nitrofurantoin for *E. coli* isolates overall and among the drug-resistant phenotypes (Table S6). Limited phenotypic data for the other baseline uropathogens in the micro-ITT NTF-S population prevented treatment comparisons.

## DISCUSSION

Pooled microbiology data from the EAGLE-2 and EAGLE-3 pivotal, global, Phase 3 trials provide a contemporary, comprehensive set of prevalence data for community-acquired uUTIs in females. Per protocol, baseline urine samples were collected and cultured from all enrolled study participants. Although study inclusion and exclusion criteria had to be met for study entry (10, 18), this collection of data represents the largest and most unbiased microbiology prevalence data set for outpatient uUTIs in females, as it is not enriched for patients with a history of uUTI, drug resistance, and/or treatment failure.

The incidence of recovered baseline uropathogens for the pooled micro-ITT population was consistent with known uUTI uropathogen epidemiology. *E. coli* is the most prevalent uropathogen isolated in community-acquired uUTIs and accounts for approximately 75% of infections overall (4–7). Similarly, *E. coli* was the most common qualifying uropathogen recovered in our pooled data with an incidence of 78%. Other typical uUTI uropathogens include *K. pneumoniae*, *P. mirabilis*, and *S. saprophyticus* (1, 3), which aligns with these pooled data that had incidences of 8%, 5%, and 2%, respectively.

Resistance to current treatment options for uUTIs with resistance rates > 10% was observed in this outpatient population. For example, while resistance to nitrofurantoin was low (1%) for the qualifying baseline *E. coli* isolates, 28% were SXT-R, 28% were FQ-R, and 15% were ESBL+ (Table 1). Of the baseline qualifying *K. pneumoniae* isolates, 23% and 25% were SXT-R and nitrofurantoin resistant, respectively, and 16% were ESBL+. Twenty-five percent of baseline qualifying *P. mirabilis* isolates were SXT-R, and all are intrinsically resistant to nitrofurantoin (19). These observations underscore the continued need for novel outpatient antibacterial options to treat uUTIs.

In addition to nitrofurantoin resistance rates displayed among the baseline qualifying uropathogens (1%–25%) and known intrinsic resistance to nitrofurantoin for several uropathogen bacterial species, a considerable percentage (15%) of participants in the pooled micro-ITT population had a baseline qualifying uropathogen that was not susceptible to nitrofurantoin, albeit the uropathogens were mostly species other than *E. coli*, which had a low resistant rate to nitrofurantoin (Table 1). This represents an unmet need in a subset of patients with uUTI who may receive nitrofurantoin as a first-line agent.

For *E. coli* and *S. saprophyticus* isolates, the drug-resistant profiles of antibacterials from these pooled EAGLE-2 and EAGLE-3 outpatient uUTI trials conducted from 2019 to 2022 were similar to those reported in a global surveillance study of UTI uropathogens with isolates collected from 2019 to 2020 in both outpatient (68.4%) and inpatient (31.6%) settings (15). Comparisons to the 2019 to 2020 surveillance study for other uropathogens recovered in the pooled trial data cannot be made, as uropathogens other than *E. coli* and *S. saprophyticus* were not assessed in the surveillance study. *E. coli* drug-resistant phenotypes recovered, and resistant rates observed, were similar between the pooled trial data (1,159 isolates) and the surveillance study (3,560 isolates) for amoxicillin-clavulanate (4% versus 5.7%), fluoroquinolones (25% versus 25.3% [ciprofloxacin]), SXT (28% versus 31.8%), and nitrofurantoin (1% versus 1.3%) (Table 1) (15). In addition, gepotidacin MIC ranges were the same (≤0.03 to 32 µg/mL) against *E. coli* isolates and drug-resistant *E. coli* phenotypes between the pooled trial data and the 2019 to 2020 surveillance study results (15). Gepotidacin MIC ranges were also similar against *S. saprophyticus* isolates for the pooled data (0.06–0.5 µg/mL) compared with the surveillance data (≤0.03 to 0.25 µg/mL) (15).

Comparison of pooled EAGLE-2 and EAGLE-3 *E. coli* isolates (*n* = 1,159 isolates from 2019 to 2022) with data from a retrospective, multicenter, cohort study of *E. coli* isolates from US outpatient female urine cultures (*n* = 1,513,882 *E. coli* isolates from 2011 to 2019) (7) demonstrated similar rates for SXT resistance (28% [Table 1] and 25.39%, respectively). The incidence of fluoroquinolone-resistant *E. coli* phenotypes was 28% in these pooled data, which was higher than the proportion of non-susceptible fluoroquinolone isolates (21.10%) previously reported (7). In the pooled data, 6% of *E. coli* isolates were MDR (combined FQ-R, SXT-R, and ESBL+; Table 1), which was higher than observed in the retrospective study, where 3.81% of *E. coli* isolates were reported as not susceptible to three or more of the following: fluoroquinolones, SXT, nitrofurantoin, and ESBL+.

There were several microbiological drug-resistant phenotype findings of interest. A higher-than-expected *E. coli* ESBL+ recovery rate of 15% for pooled data and combined treatment groups was identified (Table 1). Recently reported outpatient ESBL+ *E. coli* rates were 6.4% for US isolates collected from 2011 to 2019 (7) and 12.4% for global isolates collected from 2019 to 2021 (23). However, the increase in ESBL+ *E. coli* appears to be consistent with the model-predicted average yearly increase of 7.7% that could be expected (7) and may also be attributed to geographical differences. Also, across the range of uropathogens recovered in an outpatient setting, several drug-resistant phenotypes were observed for antibacterials that are not typical or recommended uUTI treatments, including aminoglycosides and intravenous cephalosporins (Table 1). This finding highlights the importance of antibiotic stewardship to prevent collateral damage and to ensure there are viable infectious disease treatment options in the future (24).

In the pooled EAGLE-2 and EAGLE-3 trial data sets, therapeutic, clinical, and microbiological success rates at TOC were similar across phenotypic subcategories of each species, although sample sizes were small for some *E. coli* and all *K. pneumoniae* and *P. mirabilis* phenotypic subcategories in the micro-ITT population (Fig. 1). Success rates were generally higher for gepotidacin compared with nitrofurantoin in the micro-ITT, which was not unexpected because this population included isolates not susceptible to nitrofurantoin, especially for *K. pneumoniae* and *P. mirabilis*. Outcomes in study participants with nitrofurantoin-resistant *E. coli* in the micro-ITT population were not evaluated due to the small number of isolates recovered (<10 per treatment arm). Among participants with nitrofurantoin-resistant *K. pneumoniae*, the observation of similar therapeutic success rates between treatment arms was unexpected (5/13 [38.5%] gepotidacin; 6/15 [40.0%] nitrofurantoin). A contributing factor was the unusually high clinical success rate in the nitrofurantoin arm (73.3%), which was higher than the microbiological eradication rate for nitrofurantoin-resistant *K. pneumoniae* (46.7%) and was also higher than the clinical success rate for *K. pneumoniae* overall (50.9%). The reason for this observation is not clear, though it is noted that the size of this nitrofurantoin-resistant subgroup was small.

The FDA recently approved gepotidacin for the treatment of female adult and pediatric patients 12 years of age and older weighing at least 40 kg with uUTIs caused by the following susceptible microorganisms based on *in vitro* and clinical efficacy data: *E. coli*, *K. pneumoniae*, *C. freundii* complex, *S. saprophyticus*, and *E. faecalis* (17). Other baseline uropathogens recovered in the EAGLE-2 and EAGLE-3 pooled trial data, such as *C. koseri*, *K. aerogenes*, *K. oxytoca/Raoultella ornithinolytica*, *M. morganii*, *P. mirabilis*, and *Providencia rettgeri*, were included in the prescribing details as having *in vitro* MIC_90_ values less than or equal to the Enterobacterales-susceptible breakpoint for gepotidacin (≤16 µg/mL); however, clinical efficacy had not been established in the primary population (i.e., micro-ITT NTF-S population).

The active comparator in the two gepotidacin uUTI pivotal Phase 3 trials was nitrofurantoin, which is a first-line treatment for uUTIs globally (1, 25). As such, baseline qualifying uropathogens susceptible to nitrofurantoin were required for inclusion in the primary analysis population (10). A broader range of uropathogens and gepotidacin’s activity against them, including nitrofurantoin-resistant phenotypes, has been presented herein by utilizing a pooled micro-ITT population. In addition, a notable proportion of drug-resistant phenotypes were recovered in EAGLE-2 and EAGLE-3, which may limit the use of other recommended antimicrobial agents for uUTI. Per FDA gepotidacin breakpoints and interpretive criteria (22), there was no resistance among Enterobacterales, *S. saprophyticus*, or *E. faecalis* uropathogens; the vast majority of Enterobacterales isolates were susceptible (MICs ≤ 16 µg/mL) and none were resistant (MIC ≥64 µg/mL) to gepotidacin (Table 2). For all uropathogen drug-resistant phenotypes recovered in these two trials, gepotidacin MIC_90_ values were similar (i.e., lower, equal to, or 1-dilution higher) when compared with the MIC_90_ of the overall species, which demonstrates the *in vitro* activity of gepotidacin. The overall prevalence of uropathogens resistant to ≥1 class of antimicrobial agents prescribed for uUTIs, together with the consistent efficacy of gepotidacin observed across these drug-resistant subgroups, suggests this first-in-class, oral, triazaacenaphthylene antibiotic may provide a new treatment option for uUTIs (10, 17).

## MATERIALS AND METHODS

### Study overview

The EAGLE-2 and EAGLE-3 trials were Phase 3 evaluations that compared the efficacy and safety of gepotidacin versus nitrofurantoin for the treatment of uUTI. The trial design, inclusion and exclusion criteria for participant entry, stratification, and randomization to study treatment (1:1 ratio to oral gepotidacin or oral nitrofurantoin), and ethical standards for study conduct have been described previously (10, 18). Time points for clinical and microbiological efficacy and safety assessments were Baseline (day 1; pretreatment), On-therapy (days 2–4), TOC (days 10–13), and FU (days 28 ± 3).

### Microbiological assessments

All microbiological analyses were performed at the central laboratory (PPD Laboratory Services Central Lab, Highland Heights, KY, USA), unless otherwise specified.

Participants provided a midstream clean-catch urine sample at each visit using a BD Vacutainer C&S Cup Kit (Becton Dickinson, Franklin Lakes, NJ, USA) supplied to study sites by the central laboratory. At baseline, the urine collection was performed before administration of the study treatment.

Quantitative urine culture and uropathogen identification were performed using standard methods. Urine samples were processed for culture using a sterile disposable, calibrated plastic loop with a volume capacity of 1 µL. Isolates were identified to the genus and species level by matrix-assisted laser desorption/ionization time-of-flight mass spectrometry.

Antimicrobial susceptibility testing was conducted by broth microdilution MIC methods according to CLSI procedures using Sensititre custom-dried panels (ThermoFisher Scientific, Waltham, MA, USA) at the central laboratory (19, 26). Isolates that were either nitrofurantoin-intermediate or nitrofurantoin-resistant per the central laboratory were confirmed by repeat reference broth microdilution method at Element Iowa City (JMI Laboratories; North Liberty, IA, USA). Confirmatory nitrofurantoin susceptibility testing was not performed on uropathogens that are intrinsically resistant to nitrofurantoin (19). Fosfomycin susceptibility was tested by agar dilution, according to CLSI guidelines and interpretations by Element Iowa City (19, 26). Disk diffusion testing was performed for cefadroxil, mecillinam, nitroxoline, and nitrofurantoin for all Gram-negative species; nitroxoline, penicillin, and nitrofurantoin for *E. faecalis* and *E. faecium*; and nitroxoline, penicillin, and nitrofurantoin for *S. saprophyticus* according to CLSI guidelines. For nitroxoline and cefadroxil, which do not have approved CLSI breakpoints, susceptibility interpretations were determined using European Committee on Antimicrobial Susceptibility Testing (EUCAST) guidelines (21). Gepotidacin susceptibility interpretations were not available at the time of the study but are briefly described in the text according to recently published FDA breakpoints (22). Aztreonam, cefotaxime, cefpodoxime, and ceftazidime were tested for ESBL detection according to CLSI guidelines (19). Isolates resistant to levofloxacin or ciprofloxacin were summarized as FQ-R. Isolates designated as MDR were resistant to ≥3 relevant antibiotic classes.

### Statistical analysis

Baseline qualifying uropathogens were determined by an algorithm (10) for inclusion in the efficacy analyses.

The ITT, micro-ITT, and micro-ITT NTF-S populations were previously defined herein or elsewhere (10). Baseline qualifying uropathogens were required to be susceptible to nitrofurantoin in the micro-ITT NTF-S population, whereas this was not a requirement in the micro-ITT population. Of note, uropathogens with intermediate, resistant, or no interpretation were considered not susceptible to nitrofurantoin. The ME-TOC and ME-FU populations are defined in Tables S4 and S5, respectively.

Statistical analyses were performed using SAS. Therapeutic, clinical, and microbiological outcome and response (success or failure) definitions for TOC and FU (i.e., assessment of sustained response), as previously defined (10), were determined programmatically.

Counts and percentage of participants with therapeutic (participant level), clinical (participant level), and microbiological (pathogen level) success, along with the 95% Exact Clopper-Pearson confidence intervals for the success rates, were determined for each uropathogen and drug-resistant phenotype by treatment group. No inferential testing was performed for uropathogens or drug-resistant phenotype subgroups; comparisons were based on descriptive data and numerical comparisons, not statistical testing.

For the incidence of each phenotypic subcategory for each uropathogen, percentages were calculated using the number of isolates in each phenotypic category as the numerator and the number of isolates for each respective baseline qualifying uropathogen species (present at ≥10^5^ CFU/mL) as the denominator in a post hoc analysis. Antimicrobial susceptibility results by overall frequency distribution of gepotidacin MICs, MIC_50_, and MIC_90_ were summarized by uropathogen, including for drug-resistant phenotypes.

## Data Availability

Anonymized individual participant data and study documents can be requested for further research from https://www.gsk-studyregister.com/en/.

## SUPPLEMENTAL MATERIAL

Supplemental material aac.01640-24-s0001.docx Tables S1 to S6.

ASM does not own the copyrights to Supplemental Material that may be linked to, or accessed through, an article. The authors have granted ASM a non-exclusive, world-wide license to publish the Supplemental Material files. Please contact the corresponding author directly for reuse.
