# Supplementary material for: Efficacy and in vitro activity of gepotidacin against bacterial uropathogens, including drug-resistant phenotypes, in females with uncomplicated urinary tract infections: results from two global, pivotal, phase 3 trials (EAGLE-2 and EAGLE-3)
Source: Antimicrob Agents Chemother. 2025 Sep 9;69(10):e01640-24. doi: 10.1128/aac.01640-24 (PMC12486842; doi:10.1128/aac.01640-24)
Supplement: Supplemental material — Tables S1 to S6. [file aac.01640-24-s0001.docx]

**Supplemental Material**

**Efficacy and *In Vitro* Activity of Gepotidacin Against Bacterial Uropathogens, Including Drug‑Resistant Phenotypes, in Females with Uncomplicated Urinary Tract Infections: Results from 2 Global, Pivotal, Phase 3 Trials (EAGLE‑2 and EAGLE‑3)**

Nicole E. Scangarella-Oman, Deborah L. Butler, John Breton, Derrek Brown, Cara Kasapidis, Helen Millns, Chun Huang, Caroline R. Perry, Amanda J. Sheets, Jeremy Dennison, Salim Janmohamed

**Supplemental Material Contents:**

**Table S1** Incidence of bacterial species recovered at baseline for pooled EAGLE-2 and EAGLE‑3 data (ITT population)

**Table S2** Incidence of baseline qualifying uropathogens recovered and selected drug-resistant phenotypes for pooled EAGLE-2 and EAGLE-3 data (micro-ITT NTF-S population)

**Table S3** Gepotidacin MIC frequency distribution against selected baseline qualifying uropathogens and drug-resistant phenotypes with *n* ≥10 isolates across both treatment groups for pooled EAGLE-2 and EAGLE-3 data (micro-ITT NTF-S population)

**Table S4** Therapeutic, clinical, and microbiological success at TOC by selected baseline qualifying uropathogens and drug-resistant phenotypes with *n* ≥10 participants for at least 1 treatment group for pooled EAGLE-2 and EAGLE-3 data (ME-TOC population)

**Table S5** Therapeutic, clinical, and microbiological success at FU by selected baseline qualifying uropathogens and drug-resistant phenotypes with *n* ≥10 participants for at least 1 treatment group for pooled EAGLE-2 and EAGLE-3 data (ME-FU population)

**Table S6** Therapeutic, clinical, and microbiological success at TOC by selected baseline qualifying uropathogens and drug-resistant phenotypes with *n* ≥10 participants for at least 1 treatment group for pooled EAGLE-2 and EAGLE-3 data (micro-ITT NTF-S population)

**References**

**T****able S1** Incidence of bacterial species recovered at baseline for pooled EAGLE-2 and EAGLE‑3 data (ITT population)

| **Bacterial species recovered** | **Treatment group** |  | **Total n (%)*^a^***  **N=3,136** |
| --- | --- | --- | --- |
|  | **Gepotidacin n (%)*^a^***  **N=1,572** | **Nitrofurantoin n (%)*^a^***  **N=1,564** |  |
| Total number of uropathogens recovered | 1,227 | 1,177 | 2,404 |
|  |  |  |  |
| *Escherichia coli* | 870 (71) | 831 (71) | 1,701 (71) |
| *Klebsiella pneumoniae* | 91 (7) | 106 (9) | 197 (8) |
| *Klebsiella oxytoca/Raoultella ornithinolytica* | 11 (<1) | 9 (<1) | 20 (<1) |
| *Klebsiella aerogenes* | 5 (<1) | 10 (<1) | 15 (<1) |
| *Klebsiella variicola* | 4 (<1) | 5 (<1) | 9 (<1) |
| *Proteus mirabilis* | 87 (7) | 88 (7) | 175 (7) |
| *Proteus vulgaris* group | 3 (<1) | 3 (<1) | 6 (<1) |
| *Enterobacter cloacae* complex | 14 (1) | 16 (1) | 30 (1) |
| *Citrobacter freundii* complex | 18 (1) | 13 (1) | 31 (1) |
| *Citrobacter koseri* | 12 (<1) | 15 (1) | 27 (1) |
| *Citrobacter amalonaticus* group | 2 (<1) | 1 (<1) | 3 (<1) |
| *Morganella morganii* | 23 (2) | 17 (1) | 40 (2) |
| *Providencia rettgeri* | 4 (<1) | 4 (<1) | 8 (<1) |
| *Providencia stuartii* | 3 (<1) | 1 (<1) | 4 (<1) |
| *Pantoea agglomerans* | 2 (<1) | 0 (0) | 2 (<1) |
| *Serratia marcescens* | 3 (<1) | 6 (<1) | 9 (<1) |
| *Serratia liquefaciens* | 1 (<1) | 1 (<1) | 2 (<1) |
| *Hafnia alvei* | 0 (0) | 1 (<1) | 1 (<1) |
| *Leclercia adecarboxylata* | 0 (0) | 1 (<1) | 1 (<1) |
| *Pantoea* spp. | 1 (<1) | 0 (0) | 1 (<1) |
| *Pseudomonas aeruginosa* | 12 (<1) | 14 (1) | 26 (1) |
| *Pseudomonas putida* group | 5 (<1) | 1 (<1) | 6 (<1) |
| *Pseudomonas fluorescens* group | 1 (<1) | 0 (0) | 1 (<1) |
| *Pseudomonas* spp. | 1 (<1) | 2 (<1) | 3 (<1) |
| *Stenotrophomonas maltophilia* | 5 (<1) | 1 (<1) | 6 (<1) |
| *Acinetobacter baumannii nosocomialis* group | 3 (<1) | 2 (<1) | 5 (<1) |
| *Acinetobacter pittii* | 3 (<1) | 0 (0) | 3 (<1) |
| *Acinetobacter ursingii* | 2 (<1) | 0 (0) | 2 (<1) |
| *Acinetobacter lwoffii* | 1 (<1) | 0 (0) | 1 (<1) |
| *Acinetobacter* spp. | 1 (<1) | 0 (0) | 1 (<1) |
| *Weeksella virosa* | 2 (<1) | 1 (<1) | 3 (<1) |
| *Alcaligenes faecalis* | 0 (0) | 2 (<1) | 2 (<1) |
| *Wautersiella falsenii* | 1 (<1) | 0 (0) | 1 (<1) |
| *Staphylococcus saprophyticus* | 21 (2) | 19 (2) | 40 (2) |
| *Enterococcus faecalis* | 14 (1) | 7 (<1) | 21 (<1) |
| *Enterococcus faecium* | 1 (<1) | 0 (0) | 1 (<1) |
| Sample collection details (e.g., date range, countries) have been previously described (1).  *^a^*Percentages were calculated using the total number of uropathogens at Baseline (n) as the denominator. Both qualifying and nonqualifying regulatory-approved uropathogens are presented. A participant with more than 1 uropathogen of the same species was counted multiple times. | | | |

**Table S2** Incidence of baseline qualifying uropathogens recovered and selected drug-resistant phenotypes for pooled EAGLE-2 and EAGLE-3 data (micro-ITT NTF-S population)

| **Qualifying uropathogen/phenotype or combined phenotypes***^b^* | **Treatment group** | | | **Total n (%)*^a^***  **N=1,201** |
| --- | --- | --- | --- | --- |
|  | **Gepotidacin n (%)*^a^***  **N=628** | **Nitrofurantoin n (%)*^a^***  **N=573** | |  |
| Total number of qualifying uropathogens recovered | 642 | | 583 | 1,225 |
|  |  | |  |  |
| *Escherichia coli* | 573 (89) | | 524 (90) | 1,097 (90) |
| Amoxicillin-clavulanic acid-resistant*^c^* | 29 (5) | | 16 (3) | 45 (4) |
| Ampicillin-resistant | 273 (48) | | 237 (45) | 510 (46) |
| Cefadroxil-resistant*^d^* | 83 (14) | | 62 (12) | 145 (13) |
| Cefazolin-resistant*^e^* | 97 (17) | | 73 (14) | 170 (15) |
| Ceftolozane-tazobactam-resistant | 4 (1) | | 3 (1) | 7 (1) |
| Ceftriaxone-resistant | 75 (13) | | 60 (11) | 135 (12) |
| Fosfomycin-resistant*^f^* | 5 (1) | | 4 (1) | 9 (1) |
| FQ-R*^g^* | 166 (29) | | 128 (24) | 294 (27) |
| Gentamicin-resistant | 50 (9) | | 46 (9) | 96 (9) |
| Mecillinam-resistant*^h^* | 13 (2) | | 12 (2) | 25 (2) |
| Nitroxoline-resistant*^i^* | 12 (2) | | 10 (2) | 22 (2) |
| Piperacillin-tazobactam-resistant | 12 (2) | | 8 (2) | 20 (2) |
| SXT-R | 161 (28) | | 134 (26) | 295 (27) |
| ESBL+*^j^* | 84 (15) | | 65 (12) | 149 (14) |
| MDR*^k^* | 161 (28) | | 127 (24) | 288 (26) |
| Fosfomycin-resistant*^f^* and ESBL+ *^j^* | 3 (1) | | 2 (<1) | 5 (<1) |
| FQ-R*^g^* and ESBL+ *^j^* | 61 (11) | | 42 (8) | 103 (9) |
| FQ-R*^g^* and ESBL+ *^j^* and SXT-R | 31 (5) | | 23 (4) | 54 (5) |
| FQ-R*^g^* and SXT-R | 71 (12) | | 60 (11) | 131 (12) |
| SXT-R and ESBL+*^j^* | 47 (8) | | 31 (6) | 78 (7) |
|  |  | |  |  |
| *Klebsiella pneumoniae* | 15 (2) | | 17 (3) | 32 (3) |
| Amoxicillin-clavulanic acid-resistant*^c^* | 2 (13) | | 0 | 2 (6) |
| Cefadroxil-resistant*^d^* | 2 (13) | | 3 (18) | 5 (16) |
| Cefazolin-resistant*^e^* | 2 (13) | | 3 (18) | 5 (16) |
| Ceftriaxone-resistant | 1 (7) | | 3 (18) | 4 (13) |
| FQ-R*^g^* | 2 (13) | | 6 (35) | 8 (25) |
| Gentamicin-resistant | 1 (7) | | 2 (12) | 3 (9) |
| Piperacillin-tazobactam-resistant | 2 (13) | | 1 (6) | 3 (9) |
| SXT-R | 4 (27) | | 3 (18) | 7 (22) |
| ESBL+*^j^* | 2 (13) | | 3 (18) | 5 (16) |
| MDR*^k^* | 3 (20) | | 4 (24) | 7 (22) |
| FQ-R*^g^* and ESBL+*^j^* | 2 (13) | | 3 (18) | 5 (16) |
| FQ-R*^g^* and ESBL+*^j^* and SXT-R | 0 | | 1 (6) | 1 (3) |
| FQ-R*^g^* and SXT-R | 0 | | 1 (6) | 1 (3) |
| SXT-R and ESBL+*^j^* | 0 | | 1 (6) | 1 (3) |
|  |  | |  |  |
| *Klebsiella oxytoca/Raoultella ornithinolytica* | 4 (<1) | | 4 (<1) | 8 (<1) |
| Cefazolin-resistant | 3 (75) | | 3 (75) | 6 (75) |
| MDR*^k^* | 1 (25) | | 1 (25) | 2 (25) |
|  |  | |  |  |
| *Klebsiella aerogenes* | 2 (<1) | | 3 (<1) | 5 (<1) |
|  |  | |  |  |
| *Klebsiella variicola* | 1 (<1) | | 0 | 1 (<1) |
| *Enterobacter cloacae* complex | 1 (<1) | | 4 (<1) | 5 (<1) |
| Ceftriaxone-resistant | 0 | | 1 (25) | 1 (20) |
| Piperacillin-tazobactam-resistant | 0 | | 1 (25) | 1 (20) |
| MDR*^k^* | 1 (100) | | 4 (100) | 5 (100) |
|  |  | |  |  |
| *Citrobacter freundii* complex | 12 (2) | | 5 (<1) | 17 (1) |
| Amoxicillin-clavulanic acid-resistant*^c^* | 6 (50) | | 1 (20) | 7 (41) |
| Ampicillin-resistant | 7 (58) | | 0 | 7 (41) |
| Ceftolozane-tazobactam-resistant | 2 (17) | | 0 | 2 (12) |
| Ceftriaxone-resistant | 4 (33) | | 0 | 4 (24) |
| FQ-R*^g^* | 1 (8) | | 0 | 1 (6) |
| Piperacillin-tazobactam-resistant | 3 (25) | | 0 | 3 (18) |
| SXT-R | 3 (25) | | 1 (20) | 4 (24) |
| MDR*^k^* | 7 (58) | | 0 | 7 (41) |
| FQ-R*^g^* and SXT-R | 1 (8) | | 0 | 1 (6) |
|  |  | |  |  |
| *Citrobacter koseri* | 2 (<1) | | 5 (<1) | 7 (<1) |
|  |  | |  |  |
| *Citrobacter amalonaticus* group | 2 (<1) | | 0 | 2 (<1) |
| Cefazolin-resistant | 1 (50) | | 0 | 1 (50) |
| FQ-R*^g^* | 1 (50) | | 0 | 1 (50) |
|  |  | |  |  |
| *Staphylococcus saprophyticus* | 15 (2) | | 14 (2) | 29 (2) |
| Ampicillin-resistant | 4 (27) | | 4 (29) | 8 (28) |
| Methicillin-resistant | 3 (20) | | 1 (7) | 4 (14) |
| Penicillin-resistant | 9 (60) | | 8 (57) | 17 (59) |
| MDR*^k^* | 0 | | 1 (7) | 1 (3) |
|  |  | |  |  |
| *Enterococcus faecalis* | 14 (2) | | 7 (1) | 21 (2) |
| Fosfomycin-resistant*^f^* | 1 (7) | | 0 | 1 (5) |
| FQ-R*^g^* | 4 (29) | | 2 (29) | 6 (29) |
| Penicillin-resistant | 4 (29) | | 1 (14) | 5 (24) |
| MDR*^k^* | 3 (21) | | 1 (14) | 4 (19) |
| FQ-R*^g^* and SXT-R | 4 (29) | | 2 (29) | 6 (29) |
|  |  | |  |  |
| *Enterococcus faecium* | 1 (<1) | | 0 | 1 (<1) |

*^a^*Percentage of each qualifying uropathogen was calculated using the total number of baseline qualifying uropathogens at baseline as the denominator. Percentage of each phenotypic subcategory was calculated using the number of each respective baseline qualifying uropathogen as the denominator in a posthoc analysis.

*^b^*Baseline qualifying (≥10^5^ CFU/mL) uropathogens and selected drug-resistant phenotypes are presented. All drug-resistant phenotypes were determined per CSLI or EUCAST guidelines as described in the footnotes. Phenotypes were determined per CLSI M100 2022 guidelines (2) with the exception of cefadroxil and nitroxoline, which were determined by EUCAST 2022 guidelines (3). Uropathogens and phenotypes (e.g., ampicillin‑resistant *K. pneumoniae*; amoxicillin-clavulanic acid‑resistant, ampicillin-resistant, cefazolin-resistant, and MDR *E. cloacae* complex; cefazolin‑resistant *C. freundii* complex) that are 100% resistant due to intrinsic resistance of the organism to the drug per CLSI M100 2022 guidelines are not presented. Isolates with intermediate susceptibility interpretations were not included in the corresponding drug-resistant phenotypic categories.

*^c^*Breakpoints for therapy of uUTIs; tested according to CLSI guidelines in a 2:1 ratio.

*^d^*Tested by disk diffusion method; breakpoints and interpretations per EUCAST guidelines for uUTI only.

*^e^*Breakpoints for when used as a surrogate test for oral cephalosporins for therapy of uUTIs due to *E. coli* and *K. pneumoniae*.

*^f^*Tested by agar dilution method using media supplemented with 25 *µ*g/mL of glucose-6-phosphate; breakpoints apply only to urinary tract isolates.

*^g^*FQ-R indicates resistance to ciprofloxacin and/or levofloxacin using CLSI breakpoints.

*^h^*Tested by disk diffusion method; interpretations per CLSI guidelines for testing and reporting of *E. coli* urinary tract isolates only.

*^i^*Tested by disk diffusion method; breakpoints and interpretations per EUCAST guidelines for uUTIs only due to *E. coli*.

*^j^*ESBL+ indicates extended spectrum *ꞵ*-lactamase production per CLSI M100 Table 3A.

*^k^*MDR was defined as resistance to ≥3 relevant antibacterial classes.

**Table S3** Gepotidacin MIC frequency distribution against selected baseline qualifying uropathogens and drug-resistant phenotypes with *n* ≥10 isolates across both treatment groups for pooled EAGLE-2 and EAGLE-3 data (micro-ITT NTF-S population)

| **Uropathogen/Phenotypic subcategory*^a^*** | **No. of isolates*^b^*** | **No. of isolates/(cumulative %) inhibited with gepotidacin MIC (*µ*g/mL) of*^c^*** | | | | | | | | | | | **MIC_50_ *µ*g/mL** | **MIC_90_ *µ*g/mL** |
| --- | --- | --- | --- | --- | --- | --- | --- | --- | --- | --- | --- | --- | --- | --- |
|  |  | **≤0.03** | **0.06** | **0.12** | **0.25** | **0.5** | **1** | **2** | **4** | **8** | **16** | **32** |  |  |
| *Escherichia coli* | 1,097 | 1  (<0.1) | 0  (<0.1) | 2  (0.3) | 10 (1.2) | 96 (9.9) | 480 (53.7) | 397 (89.9) | 87 (97.8) | 16 (99.3) | 7 (>99.9) | 1  (100) | 1 | 4 |
| Amoxicillin-clavulanic acid-resistant*^d^* | 45 |  |  |  |  | 3  (6.7) | 16 (42.2) | 15 (75.6) | 9 (95.6) | 1 (97.8) | 1  (100) |  | 2 | 4 |
| Ampicillin-resistant | 510 |  |  | 2  (0.4) | 8  (2.0) | 47 (11.2) | 220 (54.3) | 168 (87.3) | 47 (96.5) | 11 (98.6) | 6 (99.8) | 1  (100) | 1 | 4 |
| Cefadroxil-resistant*^e^* | 145 |  |  | 1  (0.7) | 2  (2.1) | 16 (13.1) | 49 (46.9) | 49 (80.7) | 17 (92.4) | 8 (97.9) | 3  (100) |  | 2 | 4 |
| Cefazolin-resistant*^f^* | 170 |  |  | 2  (1.2) | 2  (2.4) | 16 (11.8) | 62 (48.2) | 58 (82.4) | 19 (93.5) | 7 (97.6) | 4  (100) |  | 2 | 4 |
| Ceftriaxone-resistant | 135 |  |  | 1  (0.7) | 2  (2.2) | 13 (11.9) | 46 (45.9) | 46 (80.0) | 16 (91.9) | 7 (97.0) | 4  (100) |  | 2 | 4 |
| FQ-R*^g^* | 294 | 1  (0.3) | 0  (0.3) | 2  (1.0) | 8  (3.7) | 56 (22.8) | 105 (58.5) | 79 (85.4) | 35 (97.3) | 5 (99.0) | 3  (100) |  | 1 | 4 |
| Mecillinam-resistant*^h^* | 25 |  |  |  |  | 1  (4.0) | 8 (36.0) | 9 (72.0) | 5 (92.0) | 1 (96.0) | 0 (96.0) | 1  (100) | 2 | 4 |
| Nitroxoline-resistant*^i^* | 22 |  |  |  |  | 1  (4.5) | 10 (50.0) | 5 (72.7) | 3 (86.4) | 3  (100) |  |  | 1 | 8 |
| SXT-R | 295 |  |  | 1  (0.3) | 4  (1.7) | 37 (14.2) | 141 (62.0) | 82 (89.8) | 21 (96.9) | 6 (99.0) | 3  (100) |  | 1 | 4 |
| ESBL+*^j^* | 149 |  |  | 1  (0.7) | 2  (2.0) | 15 (12.1) | 53 (47.7) | 50 (81.2) | 17 (92.6) | 7 (97.3) | 4  (100) |  | 2 | 4 |
| MDR*^k^* | 288 |  |  | 2  (0.7) | 4  (2.1) | 36 (14.6) | 115 (54.5) | 85 (84.0) | 34 (95.8) | 9 (99.0) | 3  (100) |  | 1 | 4 |
| FQ-R*^g^* and ESBL+*^j^* | 103 |  |  | 1  (1.0) | 1 (1.9) | 12 (13.6) | 33 (45.6) | 38 (82.5) | 13 (95.1) | 2 (97.1) | 3  (100) |  | 2 | 4 |
| FQ-R*^g^* and ESBL+*^j^* and SXT-R | 54 |  |  | 1  (1.9) | 1  (3.7) | 10 (22.2) | 19 (57.4) | 16 (87.0) | 5 (96.3) | 0 (96.3) | 2  (100) |  | 1 | 4 |
| FQ-R*^g^* and SXT-R | 131 |  |  | 1  (0.8) | 4  (3.8) | 24 (22.1) | 54 (63.4) | 31 (87.0) | 13 (96.9) | 2 (98.5) | 2  (100) |  | 1 | 4 |
| SXT-R and ESBL+*^j^* | 78 |  |  | 1  (1.3) | 1  (2.6) | 12 (17.9) | 29 (55.1) | 23 (84.6) | 8 (94.9) | 2 (97.48) | 2  (100) |  | 1 | 4 |
|  | | | | | | | | | | | | | | |
| *Klebsiella pneumoniae* | 32 |  |  |  |  |  | 1  (3.1) | 5 (18.8) | 17 (71.9) | 7 (93.8) | 0 (93.8) | 2  (100) | 4 | 8 |
|  | | | | | | | | | | | | | | |
| *Citrobacter freundii* complex | 17 |  |  |  |  | 1 (5.9) | 5 (35.3) | 5 (64.7) | 4 (88.2) | 2 (100) |  |  | 2 | 8 |
|  | | | | | | | | | | | | | | |
| *Staphylococcus saprophyticus* | 29 |  | 11 (37.9) | 14 (86.2) | 2 (93.1) | 2 (100) |  |  |  |  |  |  | 0.12 | 0.25 |
|  |  |  |  |  |  |  |  |  |  |  |  |  |  |  |
| *Enterococcus faecalis* | 21 |  |  |  |  | 2 (9.5) | 11 (61.9) | 6 (90.5) | 2 (100) |  |  |  | 1 | 2 |

*^a^*All drug-resistant phenotypes were determined per CSLI or EUCAST guidelines as described in the footnotes. Phenotypes were determined per CLSI M100 2022 guidelines (2) with the exception of cefadroxil and nitroxoline, which were determined by EUCAST 2022 guidelines (3). Isolates with intermediate susceptibility interpretations were not included in the corresponding drug-resistant phenotypic categories. Uropathogens and phenotypes that are 100% resistant due to intrinsic resistance of the organism to the drug per CLSI M100 2022 guidelines are not presented. *S. saprophyticus* and *E. faecalis* drug-resistant phenotypes are not presented as the only phenotypes with *n* ≥10 isolates are 100% resistant due to intrinsic resistance (fosfomycin-resistant and SXT-R, respectively).

*^b^*Number of isolates with non-missing MIC values.

*^c^*The percentage of isolates shown at each gepotidacin MIC concentration was calculated as a cumulative value in a posthoc analysis. Gepotidacin MIC values ranged from ≤0.03 to 32 *µ*g/mL; MIC columns for 64 *µ*g/mL and >64 *µ*g/mL are not shown as there were no data to present.

*^d^*Breakpoints for therapy of uUTIs; tested according to CLSI guidelines in a 2:1 ratio.

*^e^*Tested by disk diffusion method. Breakpoints and interpretations per EUCAST guidelines for uUTIs only.

*^f^*Breakpoints for when used as a surrogate test for oral cephalosporins for therapy of uUTIs due to *E. coli* and *K. pneumoniae*.

*^g^*FQ-R indicates resistance to ciprofloxacin and/or levofloxacin using CLSI breakpoints.

*^h^*Tested by disk diffusion method. Interpretations were per CLSI guidelines for testing and reporting of *E. coli* urinary tract isolates only.

*^i^*Tested by disk diffusion method. Breakpoints and interpretations per EUCAST guidelines for uUTI only due to *E. coli*.

*^j^*ESBL+ indicates extended spectrum *ꞵ*-lactamase production per CLSI M100 Table 3A.

*^k^*MDR was defined as resistance to ≥3 relevant antibacterial classes.

**Table S4** Therapeutic, clinical, and microbiological success at TOC by selected baseline qualifying uropathogens and drug-resistant phenotypes with *n* ≥10 participants for at least 1 treatment group for pooled EAGLE-2 and EAGLE-3 data (ME-TOC population)

| **Uropathogen/Phenotypic subcategory*^a^*** | **Therapeutic success  (participant-level)*^b^*** | |  | **Clinical success**  **(participant-level)*^b^*** | |  | **Microbiological success**  **(uropathogen-level)*^c^*** | |
| --- | --- | --- | --- | --- | --- | --- | --- | --- |
|  | **Gepotidacin N = 620*^d^***  **n/N1 (%)** | **Nitrofurantoin N = 596*^d^***  **n/N1 (%)** |  | **Gepotidacin N = 620*^d^***  **n/N1 (%)** | **Nitrofurantoin N = 596*^d^***  **n/N1 (%)** |  | **Gepotidacin N = 620*^d^***  **n/N1 (%)** | **Nitrofurantoin N = 596*^d^***  **n/N1 (%)** |
| *Escherichia coli* | 311/496 (62.7) | 236/481 (49.1) |  | 368/496 (74.2) | 331/481 (68.8) |  | 415/502 (82.7) | 330/486 (67.9) |
| Amoxicillin-clavulanic acid-resistant*^e^* | 12/22 (54.5) | 7/16 (43.8) |  | 17/22 (77.3) | 9/16 (56.3) |  | 18/23 (78.3) | 11/16 (68.8) |
| Ampicillin-resistant | 143/237 (60.3) | 113/222 (50.9) |  | 176/237 (74.3) | 146/222 (65.8) |  | 194/238 (81.5) | 155/222 (69.8) |
| Cefadroxil-resistant*^f^* | 48/76 (63.2) | 26/62 (41.9) |  | 59/76 (77.6) | 37/62 (59.7) |  | 62/76 (81.6) | 41/62 (66.1) |
| Cefazolin-resistant*^g^* | 55/85 (64.7) | 30/70 (42.9) |  | 68/85 (80.0) | 42/70 (60.0) |  | 69/85 (81.2) | 47/70 (67.1) |
| Ceftriaxone-resistant | 43/69 (62.3) | 22/59 (37.3) |  | 54/69 (78.3) | 33/59 (55.9) |  | 55/69 (79.7) | 38/59 (64.4) |
| FQ-R*^h^* | 80/142 (56.3) | 53/130 (40.8) |  | 104/142 (73.2) | 81/130 (62.3) |  | 112/146 (76.7) | 79/130 (60.8) |
| Mecillinam-resistant*^i^* | 8/16 (50.0) | 4/12 (33.3) |  | 9/16 (56.3) | 8/12 (66.7) |  | 13/16 (81.3) | 6/12 (50.0) |
| Nitroxoline-resistant*^j^* | 5/8 (62.5) | 6/12 (50.0) |  | 6/8 (75.0) | 8/12 (66.7) |  | 6/8 (75.0) | 9/12 (75.0) |
| SXT-R | 89/146 (61.0) | 60/127 (47.2) |  | 109/146 (74.7) | 81/127 (63.8) |  | 124/147 (84.4) | 91/129 (70.5) |
| ESBL+*^k^* | 48/74 (64.9) | 26/64 (40.6) |  | 59/74 (79.7) | 37/64 (57.8) |  | 60/74 (81.1) | 43/64 (67.2) |
| MDR*^l^* | 86/141 (61.0) | 57/126 (45.2) |  | 107/141 (75.9) | 78/126 (61.9) |  | 113/142 (79.6) | 84/126 (66.7) |
| FQ-R*^h^* and ESBL+*^k^* | 38/56 (67.9) | 16/44 (36.4) |  | 46/56 (82.1) | 24/44 (54.5) |  | 46/56 (82.1) | 27/44 (61.4) |
| FQ-R*^h^* and ESBL+*^k^* and SXT-R | 23/32 (71.9) | 7/23 (30.4) |  | 26/32 (81.3) | 12/23 (52.2) |  | 29/32 (90.6) | 14/23 (60.9) |
| FQ-R*^h^* and SXT-R | 38/66 (57.6) | 22/59 (37.3) |  | 48/66 (72.7) | 36/59 (61.0) |  | 55/67 (82.1) | 36/59 (61.0) |
| SXT-R and ESBL+*^k^* | 30/44 (68.2) | 10/30 (33.3) |  | 35/44 (79.5) | 16/30 (53.3) |  | 38/44 (86.4) | 20/30 (66.7) |
|  |  |  |  |  |  |  |  |  |
| *Klebsiella pneumoniae* | 21/48 (43.8) | 16/43 (37.2) |  | 25/48 (52.1) | 25/43 (58.1) |  | 38/50 (76.0) | 26/44 (59.1) |
| Nitrofurantoin-resistant | 5/11 (45.5) | 6/13 (46.2) |  | 5/11 (45.5) | 10/13 (76.9) |  | 8/11 (72.7) | 7/13 (53.8) |
| SXT-R | 4/10 (40.0) | 1/6 (16.7) |  | 4/10 (40.0) | 3/6 (50.0) |  | 8/10 (80.0) | 3/6 (50.0) |
| ESBL+*^k^* | 5/10 (50.0) | 1/5 (20.0) |  | 6/10 (60.0) | 2/5 (40.0) |  | 7/10 (70.0) | 3/5 (60.0) |
| MDR*^l^* | 6/13 (46.2) | 3/10 (30.0) |  | 7/13 (53.8) | 6/10 (60.0) |  | 10/13 (76.9) | 6/10 (60.0) |
|  |  |  |  |  |  |  |  |  |
| *Proteus mirabilis* | 24/34 (70.6) | 9/30 (30.0) |  | 27/34 (79.4) | 15/30 (50.0) |  | 32/34 (94.1) | 21/30 (70.0) |
| FQ-R*^h^* | 7/11 (63.6) | 1/3 (33.3) |  | 9/11 (81.8) | 2/3 (66.7) |  | 9/11 (81.8) | 2/3 (66.7) |
| SXT-R | 8/12 (66.7) | 1/5 (20.0) |  | 10/12 (83.3) | 3/5 (60.0) |  | 10/12 (83.3) | 4/5 (80.0) |
| MDR^l^ | 11/16 (68.8) | 1/5 (20.0) |  | 13/16 (81.3) | 3/5 (60.0) |  | 14/16 (87.5) | 4/5 (80.0) |
| FQ-R*^h^* and SXT-R | 6/10 (60.0) | 1/3 (33.3) |  | 8/10 (80.0) | 2/3 (66.7) |  | 8/10 (80.0) | 2/3 (66.7) |
|  |  |  |  |  |  |  |  |  |
| *Citrobacter freundii* complex | 9/12 (75.0) | 2/6 (33.3) |  | 9/12 (75.0) | 2/6 (33.3) |  | 12/12 (100) | 6/6 (100) |

The ME-TOC population was defined as participants who were included in the micro-ITT population plus followed important components of the trial (i.e., received at least 80% of planned doses as randomized, actual treatment received was the same as the randomized treatment, had an interpretable quantitative urine culture at TOC, had evaluable clinical scores at baseline and TOC, did not receive any other systemic antimicrobials before TOC unless it was taken for the current infection, and had no other major protocol deviation that prevented evaluation of efficacy).

*^a^*All drug-resistant phenotypes were determined per CSLI or EUCAST guidelines as described in the footnotes. Phenotypes were determined per CLSI M100 2022 guidelines (2) with the exception of cefadroxil and nitroxoline, which were determined by EUCAST 2022 guidelines (3). Isolates with intermediate susceptibility interpretations were not included in the corresponding drug-resistant phenotypic categories. Only phenotypic subgroups with ≥10 participants in either treatment group based on pooled EAGLE-2 and EAGLE-3 data are presented. Therefore, there are additional phenotypes presented in the main manuscript Table 1 for incidence and Table 2 for gepotidacin *in vitro* activity due to the different threshold of ≥10 participants in the combined treatment groups presented here.

*^b^*For therapeutic and clinical response, a participant was counted once under a uropathogen category if multiple qualifying uropathogens within that category were isolated at baseline for the participant. Participants for whom all uropathogens were not eradicated and all symptoms were not resolved were considered therapeutic failures for all uropathogens. For therapeutic and clinical success: n/N1 = (n) the number of participants within the category with a response of success/(N1) the total number of participants within the category, which was the denominator for the corresponding percentages.

*^c^*For microbiological response, a participant was counted more than once under a uropathogen category if multiple qualifying uropathogens within that category were isolated at baseline for the participant. For microbiological success: n/N1 = (n) the number of isolates that are a microbiological success/(N1) the total number of isolates in the category and is the denominator for the corresponding percentages.

*^d^*The N in the header represents the total number of participants in the treatment arm.

*^e^*Breakpoints for therapy of uUTIs; tested according to CLSI guidelines in a 2:1 ratio.

*^f^*Tested by disk diffusion method. Breakpoints and interpretations per EUCAST guidelines for uUTIs only.

*^g^*Breakpoints for when used as a surrogate test for oral cephalosporins for therapy of uUTIs due to *E. coli* and *K. pneumoniae*.

*^h^*FQ-R indicates resistance to ciprofloxacin and/or levofloxacin using CLSI breakpoints.

*^i^*Tested by disk diffusion method. Interpretations were per CLSI guidelines for testing and reporting of *E. coli* urinary tract isolates only.

*^j^*Tested by disk diffusion method. Breakpoints and interpretations per EUCAST guidelines for uUTIs only due to *E. coli*.

*^k^*ESBL+ indicates extended spectrum *ꞵ*-lactamase production per CLSI M100 Table 3A.

*^l^*MDR was defined as resistance to ≥3 relevant antibacterial classes.

**Table S5** Therapeutic, clinical, and microbiological success at FU by selected baseline qualifying uropathogens and drug-resistant phenotypes with *n* ≥10 participants for at least 1 treatment group for pooled EAGLE-2 and EAGLE-3 data (ME-FU population)

| **Uropathogen/Phenotypic subcategory*^a^*** | **Therapeutic success**  **(participant-level)*^b^*** | |  | **Clinical success**  **(participant-level)*^b^*** | |  | **Microbiological success**  **(uropathogen-level)*^c^*** | |
| --- | --- | --- | --- | --- | --- | --- | --- | --- |
|  | **Gepotidacin N = 597*^d^***  **n/N1 (%)** | **Nitrofurantoin N = 581*^d^***  **n/N1 (%)** |  | **Gepotidacin N = 597*^d^***  **n/N1 (%)** | **Nitrofurantoin N = 581*^d^***  **n/N1 (%)** |  | **Gepotidacin N = 597*^d^***  **n/N1 (%)** | **Nitrofurantoin N = 581*^d^***  **n/N1 (%)** |
| *Escherichia coli* | 212/471 (44.9) | 174/474 (36.7) |  | 313/472 (66.3) | 292/474 (61.6) |  | 293/478 (61.3) | 242/479 (50.5) |
| Amoxicillin-clavulanic acid-resistant*^e^* | 7/21 (33.3) | 2/15 (13.3) |  | 12/21 (57.1) | 6/15 (40.0) |  | 10/22 (45.5) | 4/15 (26.7) |
| Ampicillin-resistant | 96/226 (42.5) | 84/223 (37.7) |  | 145/226 (64.2) | 131/223 (58.7) |  | 135/227 (59.5) | 113/223 (50.7) |
| Cefadroxil-resistant*^f^* | 28/73 (38.4) | 19/64 (29.7) |  | 46/73 (63.0) | 32/64 (50.0) |  | 43/73 (58.9) | 28/64 (43.8) |
| Cefazolin-resistant*^g^* | 33/82 (40.2) | 23/72 (31.9) |  | 54/82 (65.9) | 37/72 (51.4) |  | 48/82 (58.5) | 33/72 (45.8) |
| Ceftriaxone-resistant | 26/64 (40.6) | 16/60 (26.7) |  | 42/64 (65.6) | 28/60 (46.7) |  | 38/64 (59.4) | 25/60 (41.7) |
| FQ-R*^h^* | 48/141 (34.0) | 46/134 (34.3) |  | 89/141 (63.1) | 76/134 (56.7) |  | 73/145 (50.3) | 63/134 (47.0) |
| Mecillinam-resistant*^i^* | 5/16 (31.3) | 3/9 (33.3) |  | 7/16 (43.8) | 6/9 (66.7) |  | 10/16 (62.5) | 3/9 (33.3) |
| Nitroxoline-resistant*^j^* | 3/6 (50.0) | 5/11 (45.5) |  | 5/6 (83.3) | 8/11 (72.7) |  | 3/6 (50.0) | 7/11 (63.6) |
| SXT-R | 57/138 (41.3) | 44/127 (34.6) |  | 89/138 (64.5) | 74/127 (58.3) |  | 87/139 (62.6) | 68/129 (52.7) |
| ESBL+*^k^* | 28/71 (39.4) | 19/65 (29.2) |  | 46/71 (64.8) | 32/65 (49.2) |  | 41/71 (57.7) | 29/65 (44.6) |
| MDR*^l^* | 56/140 (40.0) | 43/128 (33.6) |  | 92/140 (65.7) | 72/128 (56.3) |  | 78/141 (55.3) | 61/128 (47.7) |
| FQ-R*^h^* and ESBL+*^k^* | 22/56 (39.3) | 14/47 (29.8) |  | 37/56 (66.1) | 23/47 (48.9) |  | 31/56 (55.4) | 21/47 (44.7) |
| FQ-R*^h^* and ESBL+*^k^* and SXT-R | 12/29 (41.4) | 6/24 (25.0) |  | 20/29 (69.0) | 13/24 (54.2) |  | 18/29 (62.1) | 11/24 (45.8) |
| FQ-R*^h^* and SXT-R | 20/61 (32.8) | 17/60 (28.3) |  | 37/61 (60.7) | 35/60 (58.3) |  | 35/62 (56.5) | 27/60 (45.0) |
| SXT-R and ESBL+*^k^* | 17/40 (42.5) | 7/29 (24.1) |  | 27/40 (67.5) | 15/29 (51.7) |  | 25/40 (62.5) | 14/29 (48.3) |
|  |  |  |  |  |  |  |  |  |
| *Klebsiella pneumoniae* | 17/47 (36.2) | 10/42 (23.8) |  | 22/47 (46.8) | 18/42 (42.9) |  | 34/49 (69.4) | 19/43 (44.2) |
| Nitrofurantoin-resistant | 3/11 (27.3) | 3/13 (23.1) |  | 5/11 (45.5) | 8/13 (61.5) |  | 6/11 (54.5) | 4/13 (30.8) |
| SXT-R | 2/10 (20.0) | 0/8 (0.0) |  | 4/10 (40.0) | 1/8 (12.5) |  | 7/10 (70.0) | 2/8 (25.0) |
| ESBL+*^k^* | 3/10 (30.0) | 1/5 (20.0) |  | 6/10 (60.0) | 1/5 (20.0) |  | 5/10 (50.0) | 2/5 (40.0) |
| MDR*^l^* | 4/13 (30.8) | 1/9 (11.1) |  | 7/13 (53.8) | 3/9 (33.3) |  | 8/13 (61.5) | 4/9 (44.4) |
|  |  |  |  |  |  |  |  |  |
| *Proteus mirabilis* | 22/34 (64.7) | 7/31 (22.6) |  | 26/34 (76.5) | 13/31 (41.9) |  | 32/34 (94.1) | 16/31 (51.6) |
| FQ-R*^h^* | 6/11 (54.5) | 1/3 (33.3) |  | 9/11 (81.8) | 2/3 (66.7) |  | 9/11 (81.8) | 1/3 (33.3) |
| SXT-R | 6/12 (50.0) | 1/5 (20.0) |  | 9/12 (75.0) | 2/5 (40.0) |  | 10/12 (83.3) | 3/5 (60.0) |
| MDR^l^ | 9/16 (56.3) | 1/5 (20.0) |  | 12/16 (75.0) | 2/5 (40.0) |  | 14/16 (87.5) | 3/5 (60.0) |
| FQ-R*^h^* and SXT-R | 5/10 (50.0) | 1/3 (33.3) |  | 8/10 (80.0) | 2/3 (66.7) |  | 8/10 (80.0) | 1/3 (33.3) |
|  |  |  |  |  |  |  |  |  |
| *Citrobacter freundii* complex | 8/12 (66.7) | 2/4 (50.0) |  | 9/12 (75.0) | 2/4 (50.0) |  | 12/12 (100) | 3/4 (75.0) |

The ME-FU population was defined similarly to the ME-TOC population (Table S45), except instead required that participants had an interpretable quantitative urine culture at FU (unless the microbiological outcome at TOC was persistence or recurrence) and evaluable clinical scores at baseline and FU (unless clinical outcome at TOC was clinical improvement or worsening).

*^a^*All drug-resistant phenotypes were determined per CSLI or EUCAST guidelines as described in the footnotes. Phenotypes were determined per CLSI M100 2022 guidelines (2) with the exception of cefadroxil and nitroxoline, which were determined by EUCAST 2022 guidelines (3). Isolates with intermediate susceptibility interpretations were not included in the corresponding drug-resistant phenotypic categories. Only phenotypic subgroups with ≥10 participants in either treatment group based on pooled EAGLE-2 and EAGLE-3 data are presented. Therefore, there are additional phenotypes presented in the main manuscript Table 1 for incidence and Table 2 for gepotidacin *in vitro* activity due to the different threshold of ≥10 participants in the combined treatment groups presented here.

*^b^*For therapeutic and clinical response, a participant was counted once under a uropathogen category if multiple qualifying uropathogens within that category were isolated at baseline for the participant. Participants for whom all uropathogens were not eradicated and all symptoms were not resolved were considered therapeutic failures for all uropathogens. For therapeutic and clinical success: n/N1 = (n) the number of participants within the category with a response of success/(N1) the total number of participants within the category, which was the denominator for the corresponding percentages.

*^c^*For microbiological response, a participant was counted more than once under a uropathogen category if multiple qualifying uropathogens within that category were isolated at baseline for the participant. For microbiological success: n/N1 = (n) the number of isolates that are a microbiological success/(N1) the total number of isolates in the category and is the denominator for the corresponding percentages.

*^d^*The N in the header represents the total number of participants in the treatment arm.

*^e^*Breakpoints for therapy of uUTIs; tested according to CLSI guidelines in a 2:1 ratio.

*^f^*Tested by disk diffusion method. Breakpoints and interpretations per EUCAST guidelines for uUTIs only.

*^g^*Breakpoints for when used as a surrogate test for oral cephalosporins for therapy of uUTIs due to *E. coli* and *K. pneumoniae*.

*^h^*FQ-R indicates resistance to ciprofloxacin and/or levofloxacin using CLSI breakpoints.

*^i^*Tested by disk diffusion method. Interpretations were per CLSI guidelines for testing and reporting of *E. coli* urinary tract isolates only.

*^j^*Tested by disk diffusion method. Breakpoints and interpretations per EUCAST guidelines for uUTI only due to *E. coli*.

*^k^*ESBL+ indicates extended spectrum *ꞵ*-lactamase production per CLSI M100 Table 3A.

*^l^*MDR was defined as resistance to ≥3 relevant antibacterial classes.

**Table S6** Therapeutic, clinical, and microbiological success at TOC by selected baseline qualifying uropathogens and drug-resistant phenotypes with *n* ≥10 participants for at least 1 treatment group for pooled EAGLE-2 and EAGLE-3 data (micro-ITT NTF-S population)

| **Uropathogen/Phenotypic subcategory*^a^*** | **Therapeutic success  (participant-level)*^b^*** | |  | **Clinical success**  **(participant-level)*^b^*** | |  | **Microbiological success**  **(uropathogen-level)*^c^*** | |
| --- | --- | --- | --- | --- | --- | --- | --- | --- |
|  | **Gepotidacin N = 628*^d^***  **n/N1 (%)** | **Nitrofurantoin N = 573*^d^***  **n/N1 (%)** |  | **Gepotidacin N = 628*^d^***  **n/N1 (%)** | **Nitrofurantoin N = 573*^d^***  **n/N1 (%)** |  | **Gepotidacin N = 628*^d^***  **n/N1 (%)** | **Nitrofurantoin N = 573*^d^***  **n/N1 (%)** |
| *Escherichia coli* | 312/566 (55.1) | 234/520 (45.0) |  | 387/566 (68.4) | 337/520 (64.8) |  | 412/573 (71.9) | 322/524 (61.5) |
| Amoxicillin-clavulanic acid-resistant*^e^* | 12/28 (42.9) | 6/16 (37.5) |  | 18/28 (64.3) | 8/16 (50.0) |  | 18/29 (62.1) | 9/16 (56.3) |
| Ampicillin-resistant | 141/272 (51.8) | 110/237 (46.4) |  | 181/272 (66.5) | 148/237 (62.4) |  | 187/273 (68.5) | 148/237 (62.4) |
| Cefadroxil-resistant*^f^* | 44/83 (53.0) | 25/62 (40.3) |  | 57/83 (68.7) | 35/62 (56.5) |  | 52/83 (62.7) | 38/62 (61.3) |
| Cefazolin-resistant*^g^* | 52/97 (53.6) | 29/73 (39.7) |  | 67/97 (69.1) | 40/73 (54.8) |  | 61/97 (62.9) | 44/73 (60.3) |
| Ceftriaxone-resistant | 40/75 (53.3) | 21/60 (35.0) |  | 52/75 (69.3) | 31/60 (51.7) |  | 47/75 (62.7) | 35/60 (58.3) |
| FQ-R*^h^* | 78/161 (48.4) | 50/128 (39.1) |  | 112/161 (69.6) | 79/128 (61.7) |  | 106/166 (63.9) | 71/128 (55.5) |
| Mecillinam-resistant*^i^* | 7/13 (53.8) | 4/12 (33.3) |  | 7/13 (53.8) | 7/12 (58.3) |  | 9/13 (69.2) | 5/12 (41.7) |
| Nitroxoline-resistant*^j^* | 6/12 (50.0) | 5/10 (50.0) |  | 7/12 (58.3) | 7/10 (70.0) |  | 7/12 (58.3) | 7/10 (70.0) |
| SXT-R | 87/160 (54.4) | 58/132 (43.9) |  | 111/160 (69.4) | 84/132 (63.6) |  | 119/161 (73.9) | 84/134 (62.7) |
| ESBL+*^k^* | 45/84 (53.6) | 25/65 (38.5) |  | 58/84 (69.0) | 35/65 (53.8) |  | 52/84 (61.9) | 40/65 (61.5) |
| MDR*^l^* | 83/160 (51.9) | 53/127 (41.7) |  | 111/160 (69.4) | 77/127 (60.6) |  | 105/161 (65.2) | 74/127 (58.3) |
| FQ-R*^h^* and ESBL+*^k^* | 35/61 (57.4) | 15/42 (35.7) |  | 44/61 (72.1) | 22/42 (52.4) |  | 39/61 (63.9) | 24/42 (57.1) |
| FQ-R*^h^* and ESBL+*^k^* and SXT-R | 20/31 (64.5) | 7/23 (30.4) |  | 23/31 (74.2) | 12/23 (52.2) |  | 22/31 (71.0) | 13/23 (56.5) |
| FQ-R*^h^* and SXT-R | 35/70 (50.0) | 21/60 (35.0) |  | 47/70 (67.1) | 39/60 (65.0) |  | 49/71 (69.0) | 32/60 (53.3) |
| SXT-R and ESBL+*^k^* | 27/47 (57.4) | 10/31 (32.3) |  | 33/47 (70.2) | 16/31 (51.6) |  | 31/47 (66.0) | 19/31 (61.3) |
|  |  |  |  |  |  |  |  |  |
| *Klebsiella pneumoniae* | 6/14 (42.9) | 6/16 (37.5) |  | 8/14 (57.1) | 9/16 (56.3) |  | 11/15 (73.3) | 9/17 (52.9) |
|  |  |  |  |  |  |  |  |  |
| *Citrobacter freundii* complex | 8/12 (66.7) | 2/5 (40.0) |  | 8/12 (66.7) | 2/5 (40.0) |  | 11/12 (91.7) | 5/5 (100) |
|  |  |  |  |  |  |  |  |  |
| *Staphylococcus saprophyticus* | 9/15 (60.0) | 11/14 (78.6) |  | 9/15 (60.0) | 13/14 (92.9) |  | 12/15 (80.0) | 12/14 (85.7) |
|  |  |  |  |  |  |  |  |  |
| *Enterococcus faecalis* | 8/14 (57.1) | 2/7 (28.6) |  | 8/14 (57.1) | 3/7 (42.9) |  | 12/14 (85.7) | 6/7 (85.7) |

*^a^*All drug-resistant phenotypes were determined per CSLI or EUCAST guidelines as described in the footnotes. Phenotypes were determined per CLSI M100 2022 guidelines (2) with the exception of cefadroxil and nitroxoline, which were determined by EUCAST 2022 guidelines (3). Isolates with intermediate susceptibility interpretations were not included in the corresponding drug-resistant phenotypic categories. Only phenotypic subgroups with ≥10 participants in either treatment group based on pooled EAGLE-2 and EAGLE-3 data are presented. Therefore, there are additional phenotypes presented in the main manuscript Table 1 for incidence and Table 2 for gepotidacin *in vitro* activity due to the different threshold of ≥10 participants in the combined treatment groups presented here.

*^b^*For therapeutic and clinical response, a participant was counted once under a uropathogen category if multiple qualifying uropathogens within that category were isolated at baseline for the participant. Participants for whom all uropathogens were not eradicated and all symptoms were not resolved were considered therapeutic failures for all uropathogens. For therapeutic and clinical success: n/N1 = (n) the number of participants within the category with a response of success/(N1) the total number of participants within the category, which was the denominator for the corresponding percentages.

*^c^*For microbiological response, a participant was counted more than once under a uropathogen category if multiple qualifying uropathogens within that category were isolated at baseline for the participant. For microbiological success: n/N1 = (n) the number of isolates that are a microbiological success/(N1) the total number of isolates in the category and is the denominator for the corresponding percentages.

*^d^*The N in the header represents the total number of participants in the treatment arm.

*^e^*Breakpoints for therapy of uUTIs; tested according to CLSI guidelines in a 2:1 ratio.

*^f^*Tested by disk diffusion method. Breakpoints and interpretations per EUCAST guidelines for uUTIs only.

*^g^*Breakpoints for when used as a surrogate test for oral cephalosporins for therapy of uUTIs due to *E. coli* and *K. pneumoniae*.

*^h^*FQ-R indicates resistance to ciprofloxacin and/or levofloxacin using CLSI breakpoints.

*^i^*Tested by disk diffusion method. Interpretations were per CLSI guidelines for testing and reporting of *E. coli* urinary tract isolates only.

*^j^*Tested by disk diffusion method. Breakpoints and interpretations per EUCAST guidelines for uUTIs only due to *E. coli*.

*^k^*ESBL+ indicates extended spectrum *ꞵ*-lactamase production per CLSI M100 Table 3A.

*^l^*MDR was defined as resistance to ≥3 relevant antibacterial classes.

**References**

1. Wagenlehner F, Perry CR, Hooton TM, Scangarella-Oman NE, Millns H, Powell M, Jarvis E, Dennison J, Sheets A, Butler D, Breton J, Janmohamed S. 2024. Oral gepotidacin versus nitrofurantoin in patients with uncomplicated urinary tract infection (EAGLE-2 and EAGLE‑3): two randomised, controlled, double-blind, double-dummy, phase 3, non‑inferiority trials. Lancet 403:741–755.
2. Clinical and Laboratory Standards Institute. 2022. Performance standards for antimicrobial susceptibility testing. M100Ed32. Clinical and Laboratory Standards Institute, Wayne, PA.
3. European Committee on Antimicrobial Susceptibility Testing. 2022. Breakpoint tables for interpretation of MICs and zone diameters, version 12.0, https://www.eucast.org/fileadmin/src/media/PDFs/EUCAST_files/Breakpoint_tables/v_12.0_Breakpoint_Tables.pdf.
